# National mortality burden attributable to the unprecedented heatwave in 2022 in China

**DOI:** 10.1186/s40779-025-00676-2

**Published:** 2025-12-15

**Authors:** Jian-Xiong Hu, Yu-Lin Zhuo, Guan-Hao He, Jiang-Mei Liu, Yan-Fang Guo, Tian-Tian Li, Wei-Wei Gong, Fang-Fang Zeng, Hai-Lai Duan, Rui-Lin Meng, Chun-Liang Zhou, Yi-Ze Xiao, Min Yu, Biao Huang, Mai-Geng Zhou, Wen-Jun Ma, Tao Liu

**Affiliations:** 1 Department of Public Health and Preventive Medicine, School of Medicine, Jinan University, Guangzhou, 510632 China; 2 Key Laboratory of Viral Pathogenesis & Infection Prevention and Control (Jinan University), Ministry of Education, Guangzhou, 510632 China; 3 Guangdong Provincial Center for Disease Control and Prevention, Guangzhou, 511430 China; 4 The National Center for Chronic and Noncommunicable Disease Control and Prevention, Beijing, 100050 China; 5 Bao’an Chronic Diseases Prevent and Cure Hospital, Shenzhen, 518100 Guangdong China; 6 Chinese Center for Disease Control and Prevention, National Institute of Environmental Health, Beijing, 100021 China; 7 Zhejiang Provincial Center for Disease Control and Prevention, Hangzhou, 310009 China; 8 Climate Center of Guangdong Province, Guangzhou, 510640 China; 9 Hunan Provincial Center for Disease Control and Prevention, Changsha, 410005 China; 10 Yunnan Provincial Center for Disease Control and Prevention, Kunming, 650034 China; 11 Jilin Provincial Center for Disease Control and Prevention, Changchun, 130062 China; 12 China Greater Bay Area Research Center of Environmental Health, School of Medicine, Jinan University, Guangzhou, 510632 China

**Keywords:** Climate change, Heatwave, Mortality, Risk, Burden

## Abstract

**Background:**

In 2022, China experienced an unprecedented heatwave event, raising concerns about the health impacts of heatwaves. This study aims to understand the devastating health risk of the exceptional heatwave in 2022 by comparing heatwave-related mortality burden in 2022 with that during 2000–2021.

**Methods:**

We collected daily mortality and daily maximum temperature (DMT) during 2006–2017 in 364 locations (counties/districts) of China. Heatwave was defined as an event with 2 or more consecutive days of DMT exceeding the 92.5th percentile. We employed a distributed lag nonlinear model (DLNM) and a meta-analysis to examine the heatwave-mortality association based on the data from 364 counties/districts, and then this association was used to assess the mortality burden attributable to heatwaves during 2000–2022 in 368 cities in China. A percentage change (%) indicator, comparing the 2022 mortality burden to the average value from 2000 to 2021, was further calculated to highlight the severity of heatwaves in 2022.

**Results:**

In the past 2 decades, the frequency and intensity of heatwaves in China significantly increased, with the cumulative excessive degree-day increasing to 31,626 in 2022 compared with the annual average value of 13,772 during 2000–2021 across China. In 2022, we observed 62,961 [95% confidence internal (CI) 54,945–70,413] heatwave-related deaths in China, which was much higher than the annual average [35,987 (95% CI 31,252–40,471)] attributable to heatwaves during 2000–2021. The vulnerability groups of heatwave-related mortality in 2022 primarily included patients with cardiovascular diseases [40,567 (95% CI 35,313–45,404)], females [35,876 (95% CI 31,035–41,005)], and people aged over 65 years [56,208 (95% CI 49,023–62,864)]; and greater heatwave-related mortality was found in eastern-central China. The attributable fraction (AF) of heatwave-related deaths increased from an annual average of 11.01‰ (95% CI 9.56–12.38) during 2000–2021 to 18.11‰ (95% CI 15.80–20.25) in 2022 with 64.43% increment (95% CI 38.10–93.78), and the increase rates were greater in Xizang Autonomous Region (159.77%, 95% CI 12.84–477.87) and Sichuan Province (133.64%, 95% CI 3.84–416.61).

**Conclusions:**

This study indicated that the frequency and intensity of heatwaves significantly increased in the past 2 decades in China, and the 2022 heatwaves were linked to a substantial mortality burden in China, with significant population and regional heterogeneity. Our findings underscore the need for developing comprehensive heat adaptation plans in the context of rapid aging and ongoing global warming.

**Supplementary Information:**

The online version contains supplementary material available at 10.1186/s40779-025-00676-2.

## Background

With climate change, extreme weather events are growing increasingly frequent and intense worldwide [1]. The Intergovernmental Panel on Climate Change (IPCC) has reported that the frequency, duration, and intensity of heatwaves have substantially increased since the 1950s, and years with exceptional heatwaves are being observed more frequently [2], such as the 2003 heatwaves in France, the 2010 heatwaves in India, and the 2011 heatwaves in Australia [3–6]. It was projected that, even if global warming stabilizes at 1.5 °C, an increasing trend in heatwaves is still inevitable in the near future [2].

In 2022, a record-breaking and large-scale heatwave event in the Northern Hemisphere affected regions and populations on an unprecedented scale. The Copernicus Climate Change Service reported that the global average temperature in August 2022 was 0.3 °C warmer than the average level during 1991–2020, making it the third hottest August on record [7]. In Europe, the summer season (June-August) in 2022 was the hottest on record, characterized by a series of intense heatwaves, and leading to drought events and wildfires [7]. Additionally, the summer heatwaves in 2022 were recorded as the most intense since 1961 in China [8], with the national average temperature reaching a record-breaking 22.3 °C and the number of hot days (daily maximum temperature ≥ 35.0 °C) reaching a high value of 16.4 d, which is 7.3 d more than the normal year and the most since 1961 [9, 10].

Heatwaves can impact human health in direct and indirect ways, contributing to increased morbidity and mortality from various causes [11–16]. A global study including 400 communities in 18 countries/regions reported that heatwaves were associated with increased risk of overall deaths, and the associations were greater in moderate cold and moderate hot areas [17]. A study in 1943 counties in the United States found that heatwaves were associated with an 18% increase in fluid and electrolyte disorders, a 14% increase in renal failure, and a 154% increase in heat stroke [18]. Moreover, the heatwave-related health risks were particularly stronger among the elderly, women, and individuals with pre-existing cardiovascular or respiratory conditions [19–22]. A recent Chinese study based on physical examination data reported that exposure to heatwaves may significantly alter clinical/subclinical cardiovascular biomarkers, including blood pressure changes, increased heart rate, acute systemic inflammation, elevated blood viscosity, and myocardial injury [23]. The previous findings highlighted the significant health impacts of heatwaves in the context of climate change.

Although the exposure-response associations between heatwaves and mortality have been estimated in many previous studies, estimating the number of deaths caused by heatwaves could provide clearer information for policy making and risk communication. For example, the 1995 heatwaves in Chicago caused 514 heat-related deaths and 696 excess deaths [24]. More than 70 thousand deaths were caused by the heat in Europe during the summer of 2003 [25]. Recently, studies have assessed the mortality burden of the 2022 heatwaves [26–29]. For example, a study covering 35 European countries estimated 61,672 deaths attributable to heat between May 30 and September 4, 2022, with the highest heat-related mortality numbers in Italy, Spain, and Germany [27]. China, as a country with a different socio-economy and climate from Europe, was also seriously attacked by the 2022 heatwaves [30]. Although previous studies have estimated the health impacts associated with heatwaves in China such as the spatiotemporal variations in heatwave-related mortality burden from 1979 to 2020 [31], and the hospitalization burden on the urinary system as well as economic losses caused by heatwaves from 2014 to 2019 [32], none of these studies covered the unprecedented 2022 heatwave. This research gap prevents a clear identification of the health burden posed by sudden and intense heatwaves, which may undermine efforts to address climate change.

In this study, we estimated the heatwave-associated mortality risk using a nationwide dataset and then compared the national mortality burden attributable to the 2022 heatwaves with the annual average of heatwave-related mortality burden during 2000–2021, which can deepen our understanding of the devastating health effects of exceptional heatwaves.

## Method

### Data sources

We collected the daily number of non-accidental mortality during 2006–2017 in 364 locations (counties/districts) across China based on death registration records, of which 294 locations in Guangdong, Zhejiang, Hunan, Jilin, and Yunnan (2013–2017) were obtained from the corresponding provincial death surveillance systems, and the left 70 locations (2006–2011) were sourced from China’s Disease Surveillance Point System (DSPS). The DSPS is administered by the Chinese Center for Disease Control and Prevention (China CDC), and the provincial death surveillance system was administered by provincial CDCs in accordance with the same technical protocols as the DSPS. At each surveillance location, all death cases certified by clinicians or professional staff from local CDCs are reported to the DSPS via direct online reporting [33]. The daily number of non-accidental mortality was used to estimate the heatwave-mortality associations via time-series analyses, a method that depends on both good quality of mortality data and adequate death count. Therefore, 364 locations included in this study met either or both criteria: 1) a population size of > 200,000; 2) an annual mortality rate of > 4‰. This is consistent with the criteria used in previous similar studies conducted in China [34, 35]. The causes of death were classified by the International Classification of Diseases, 10th Edition (ICD-10) [36]. Consistent with previous studies [16, 37], we focused on non-accidental mortality related to the physiological effects of heatwaves, including cardiovascular diseases (I00-I99), respiratory diseases (J00-J99), and other diseases (remaining codes were A00-R99). Data were also grouped by sex (male and female), age (0–64 years and ≥ 65 years), and region (Northern, Eastern, Western, and Southern).

The daily maximum temperature (°C), daily minimum temperature (°C), daily mean temperature (°C), and relative humidity (%) data were derived from the 5th generation ECMWF (European Centre for Medium-Range Weather Forecasts) atmospheric reanalysis (ERA5), which is a gridded dataset at a global-scale with a spatial resolution of 0.25° × 0.25° and an hourly temporal resolution. After our validation, the temperature data from ERA5 reanalysis displays a strong similarity to the meteorological station observations. The coefficients of determination (*R*^2^) for the daily maximum temperature, daily minimum temperature, and daily mean temperature were 0.93, 0.94, and 0.95, while the root mean square errors (RMSE) were 3.24, 3.27, and 2.87 °C, respectively. We extracted temperature data from the grids overlapping with each selected location and city in China, and then calculated the average daily temperature and relative humidity for these grids. Location-specific temperature data were used to define heatwaves and to estimate the heatwave-mortality associations, while the city-specific temperature data were used to assess the heatwave-related mortality burden. Daily concentrations of particulate matter with an aerodynamic diameter of ≤ 2.5 μm (PM_2.5_) and ozone (O_3_) during the study period were extracted from the China High Air Pollutants (CHAP) dataset (http://www.geodata.cn), a gridded dataset with a spatial resolution of 1 × 1 km. After 10-fold cross-validation, the *R*^2^ for PM_2.5_ and O_3_ were 0.92 and 0.87, respectively [38, 39].

Population data from 2000 to 2022 were obtained from The LandScan Global Population Data (https://landscan.ornl.gov/) at a 1 × 1 km grid resolution. A previous study validated its accuracy at the city level using 2018 statistical yearbook population data, confirming an excellent accuracy (*R*^2^ > 0.98) [40]. To assess the heatwave-associated mortality burden, we obtained annual national and provincial mortality rates from 2000 to 2019 from the Global Burden of Disease, Injuries, and Risk Factors Study 2023 (GBD 2023, https://www.healthdata.org/), including non-accidental mortality rates (sex and age groups) and cause-specific mortality rates. Given the potential impact of Corona Virus Disease 2019 (COVID-19) on mortality trends, we excluded data from 2020 to 2022 and assumed that post-2019 mortality rates remained stable. The gross domestic product (GDP) data of cities during the study period were collected from the statistical yearbooks.

### Definition of heatwaves and exposure assessment

Considering that heatwaves mainly occur during the warm season, our study focused on the period from May to September [41]. In our preliminary analysis, we modeled and compared the associations of 21 heatwave definitions with mortality based on data from 2006–2017 using the Akaike Information Criterion (AIC) values (Additional file 1: Table S1). Based on the lowest value of AIC, a heatwave was defined as an event in which the daily maximum temperature exceeded the threshold (92.5th percentile of daily maximum temperature distribution during 2006–2017) for 2 or more consecutive days. Furthermore, we used a composite indicator, the cumulative excessive degree-day (CEDD) [42], to comprehensively quantify the dimensions of heatwave frequency and intensity, which was defined as the sum of the temperatures above the threshold for each heatwave day:

$${\text{CEDD = }}\sum {\text{max(0, daily maximum temperature - threshold)}}$$

To more precisely characterize population exposure to heatwaves, we utilized a novel indicator, namely person-days, derived from multiplying the number of heatwave days by the number of people exposed to the heatwaves [43]. We separately estimated the person-days of heatwave exposure from 2000 to 2022 for younger people (0–64 years) and older people (≥ 65 years). In addition, we categorized all cities into high-, medium-, and low-development levels based on GDP, and further calculated population heatwave exposure for cities at different development levels.

### Statistical analysis

#### Exposure-response association between short-term heatwave exposure and mortality

To determine the relationship between short-term heatwave exposure and mortality, we employed a standard 2-stage approach using data from 364 locations during the warm seasons of 2006–2017. In the first stage, we analyzed location-specific associations between short-term heatwave exposure and mortality using a distributed lag nonlinear model (DLNM) with a quasi-Poisson distribution function. We introduced a cross-basis function to estimate exposure and lag effects [44]. Preliminary analyses showed that the effects of the heatwaves were mainly clustered in the first 3 d (Additional file 1: Fig. S1); therefore, we applied a maximum lag of 3 d to estimate the lag effects using a natural cubic spline (*ns*) with an intercept and 2 internal knots placed at equally spaced values in the log value. We used an “integer” function to estimate exposure effects, as heatwaves were treated as indicator variables of 0 and 1 [45]. Several covariates were incorporated, including PM_2.5_, O_3_, *ns* of relative humidity with 3 degrees of freedom (*df*s), *ns* of time with 4 *df*s per year to account for seasonal and long-term trends in mortality, and a categorical variable for the day-of-week to account for potential variations within a week.

In the second-stage analysis, we used a random-effects meta-analysis to estimate the pooled cumulative effect of heatwaves on mortality. The heterogeneity was tested by Cochran’s *Q* method and extension of the *I*^2^ statistic. We reported the excess risk (ER) with a 95% confidence interval (CI) for mortality due to short-term heatwave exposure. The ER was calculated as follows:

$${\text{ER}} = (e^{\beta } - 1) \times 100\%$$

where *β* represents the heatwave coefficient obtained from the 2-stage approach described above. Furthermore, we performed stratified analyses by cause-specific categories (cardiovascular diseases, respiratory diseases, and other diseases), sex (male and female), and age (0–64 years and ≥ 65 years).

#### The estimation of mortality burden attributable to heatwave

We calculated the attributable number (AN) and attributable fraction (AF) of deaths related to heatwaves during warm seasons from 2000 to 2022 at the city level (368 cities) using the following formula:

$$AN_{c,y} = \mathop \sum \limits_{i = 1}^{d} pop_{c,y} \times Mort_{c,y,i} \times HW_{c,y,i} \times \left( {1 - \frac{1}{{e^{{\beta_{c} }} }}} \right)$$

$$AF_{c,y} = AN_{c,y} /\left( {pop_{c,y} \times Mort_{c,y,s} } \right)$$

where *pop*_*c,y*_ denotes the population size of city *c* in year *y*. *Mort*_*c,y,i*_ refers to the corresponding mortality rate on day *i*. *HW*_*c,y,i*_ indicates whether day *i* is a heatwave day. *e* indicates a natural constant, and *β*_*c*_ is the modeled cumulative effect. *Mort*_*c,y,s*_ indicates the average mortality rates in the warm season of city *c* in year *y*. Then, based on the city level data, we calculated the average AN and AF.

We also compared AN and AF in 2022 with the annual average during 2000–2021 using a percentage change (%) indicator:

$${\text{Percentage}}\;{\text{change}}\,(\% ) = \frac{{{\text{Value}}_{{{2022}}} {\text{ - Average}}\;{\text{value}}_{{2000 - 2021}} }}{{{\text{Average}}\;{\text{value}}_{{2000 - 2021}} }}{\text{x}}100\%$$

In addition, the uncertainty in AN, AF, and percentage change was quantified by Monte Carlo simulations to obtain a 95% CI from 1000 samples, based on the assumption that heatwave-mortality associations and mortality rates follow a normal distribution, which has been widely adopted in previous studies [46–48]. The 95% CI was defined as the 2.5th and 97.5th percentiles of sample values.

All data were analyzed using R software (version 4.2.1). The “dlnm” and “mixmeta” packages were used to fit the model and conduct the meta-analysis, respectively. Statistical tests were two-sided, with a statistical significance level set at *P* < 0.05.

### Sensitivity analyses

A series of sensitivity analyses were conducted to test the robustness of our results, including adjustments to the heatwave definitions, lag days (1–5 d), *dfs* for time variable (3–5/year), and air pollutants (controlling only for PM_2.5_ or O_3_, or no control). We also used an autoregressive integrated moving average (ARIMA) model [49] to estimate mortality rates for 2020–2022 based on the 2000–2019 mortality rates, and applied these estimated rates for sensitivity analysis. In addition, we compared the heatwave-associated excess mortality risks between the periods of 2006–2011 and 2013–2017. Finally, we compared the risks and burdens of heatwaves defined by daily maximum temperature, daily minimum temperature, and heat index. The heat index is an indicator used to reflect the apparent temperature (or perceived temperature), which is calculated based on daily mean temperature and relative humidity [50].

## Results

### Exposure-response association of heatwaves with mortality

Our analysis encompassed 2,580,291 deaths across 364 locations in China during the warm seasons of 2006–2017. Across all study locations, the average daily mean deaths were 8, and the average daily maximum temperature was 27.9 °C (Additional file 1: Table S2). The average daily mean deaths in heatwave days were higher than those in non-heatwave days (Additional file 1: Table S3).

Heatwave exposure was associated with 8.41% (95% CI 7.44–9.39) increase in non-accidental mortality risk. Cochran’s *Q* test and *I*^2^ statistic indicate significant location heterogeneity across 364 districts/counties (*Q* = 603.69, *I*^2^ = 39.90%, *P* < 0.01). Stratified analyses showed greater associations with cardiovascular diseases (ER = 11.42%, 95% CI 9.96–12.91), females (ER = 11.69%, 95% CI 10.22–13.17), older people (≥ 65 years) (ER = 9.89%, 95% CI 8.74–11.04), and Eastern China (ER = 10.96%, 95% CI 9.60–12.34) (Table 1).

**Table 1:** Excess risk (ER) of mortality during heatwave days compared with non-heatwave days by subgroups [% (95% CI)]

Characteristics	Excess risk of mortality				
	National	Northern China	Eastern China	Western China	Southern China
Non-accidental mortality	8.41 (7.44–9.39)	8.13 (5.80–10.52)	10.96 (9.60–12.34)	4.69 (2.28–7.15)	6.66 (4.76–8.59)
Cause-specific					
Cardiovascular diseases	11.42 (9.96–12.91)	12.52 (9.44–15.69)	14.99 (12.65–17.38)	5.11 (1.38–8.97)	9.74 (7.14–12.41)
Respiratory diseases	10.48 (8.38–12.63)	6.94 (-0.22 to 14.60)	15.93 (12.76–19.20)	4.14 (-0.19 to 8.66)	7.54 (4.08–11.12)
Other diseases	5.01 (3.89–6.14)	2.06 (-1.18 to 5.40)	7.61 (6.13–9.12)	3.99 (0.78–7.32)	1.85 (-0.22 to 3.97)
Sex					
Male	5.94 (4.94–6.94)	6.58 (3.82–9.41)	7.58 (6.11–9.07)	3.76 (1.07–6.53)	4.12 (2.30–5.98)
Female	11.69 (10.22–13.17)	9.82 (6.13–13.63)	15.71 (13.64–17.82)	5.76 (1.96–9.69)	9.92 (7.24–12.67)
Age (years)					
0 – 64	3.91 (2.68–5.15)	3.09 (-0.29 to 6.58)	5.77 (3.94–7.63)	3.32 (-0.43 to 7.22)	1.49 (-0.74 to 3.78)
≥ 65	9.89 (8.74–11.04)	10.47 (7.47–13.56)	12.63 (11.05–14.22)	5.16 (2.41–7.99)	7.99 (5.73–10.30)

*CI* confidence interval

### Characteristics of heatwaves between 2000 and 2022

**Fig. 1:**
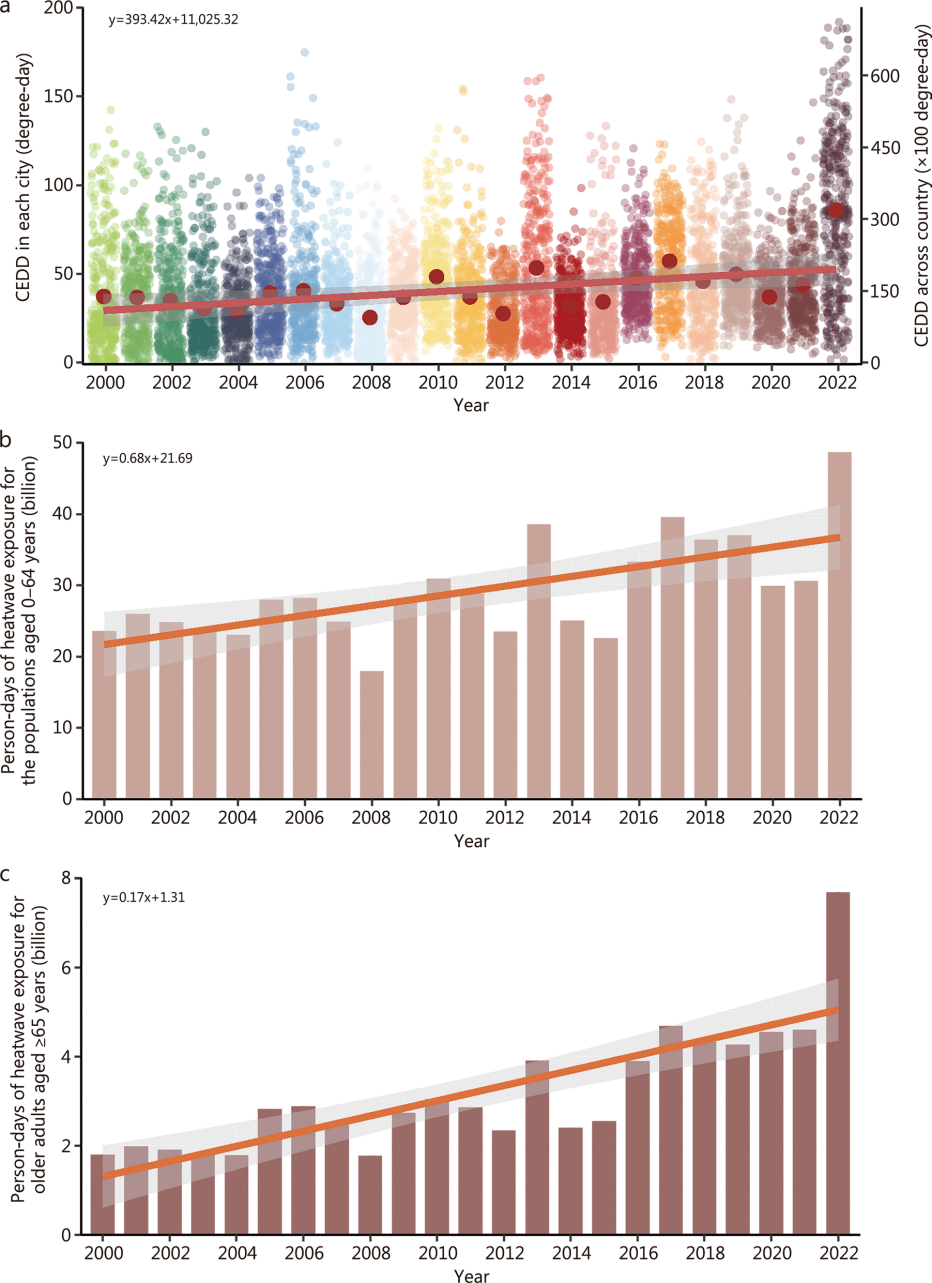
Characteristics of heatwaves occurred during 2000–2022 in China. **a** Temporal trend of cumulative excessive degree-day (CEDD) at the city level from 2000 to 2022. **b** Temporal trend of person-days of heatwave exposure for the populations aged 0–64 years from 2000 to 2022. **c** Temporal trend of person-days of heatwave exposure for older adults aged ≥ 65 years from 2000 to 2022

We observed increasing trends of number of heatwave days in each city and average daily maximum temperature during the period from 2000 to 2022 (Additional file 1: Fig. S2). Particularly, during the warm season of 2022, 2795 heatwaves were recorded at the city level across China, with an average duration of 5.09 d per heatwave and an average daily maximum temperature of 32.39 °C. These values were significantly greater than the average levels observed during 2000–2021 (2189 heatwaves, 3.95 d, and 31.74 °C, respectively), with all *P*-values < 0.001 (Additional file 1: Table S4). The annual CEDD significantly increased from 2000 to 2022, with an annual average of 13,772 degree-days during 2000–2021, and reaching a maximum of 31,626 degree-days in 2022 (Fig. 1a). Spatially, the increases in average maximum temperature, number of heatwave days, and CEDD were more pronounced in Central-Eastern China (Table 2 and Additional file 1: Table S5, S6). For example, the 3 provinces with the largest CEDD growth margins were Sichuan, Henan, and Hubei. Similarly, the number of person-days exposed to heatwaves in 2022 was also much higher than that in the annual average during 2000–2021, especially among people aged ≥ 65 years (Fig. 1b, c). Moreover, a greater increase in person-days exposure to heatwaves was observed in Central-Eastern China (Table 3). Among the provinces or municipalitys in this region, the three with the largest growth in heatwave exposure person-days among people aged ≥ 65 years were Sichuan, Henan, and Jiangsu. In addition. In addition, this exposure intensity tended to increase with the rise in the development level of cities. For example, in the high-development cities, a total of 40.57 million person-days in people ≥ 65 years were exposed to heatwaves in 2022, which was significantly greater than that in the average level during 2000–2021 [(15.12 ± 5.47) million person-days], with *P*-value < 0.001(Additional file 1: Table S7).

**Table 2:** Differences in the CEDD (degree-days) between 2022 and the annual average during 2000–2021 in China

Province/Municipality	2000–2021^a^	2022	Difference^*^	*P*-value
Sichuan	781.76	3827.13	3045.37	< 0.001
Henan	901.84	2396.54	1494.70	< 0.001
Hubei	690.28	2178.60	1488.32	< 0.001
Anhui	779.07	2130.34	1351.27	< 0.001
Jiangsu	587.43	1701.55	1114.12	< 0.001
Zhejiang	404.46	1471.22	1066.76	< 0.001
Hunan	530.75	1545.07	1014.32	< 0.001
Jiangxi	391.94	1240.47	848.53	< 0.001
Xinjiang	1133.60	1977.40	843.80	< 0.001
Guizhou	284.26	948.26	664.00	< 0.001
Gansu	669.43	1319.55	650.12	< 0.001
Shandong	766.82	1397.15	630.33	< 0.001
Qinghai	357.66	884.30	526.64	< 0.001
Shaanxi	471.56	941.44	469.88	< 0.001
Xizang	170.16	617.97	447.81	< 0.001
Guangdong	475.11	905.47	430.36	< 0.001
Fujian	241.84	662.43	420.59	< 0.001
Shanxi	514.87	892.32	377.45	< 0.001
Hebei	538.13	871.30	333.17	< 0.001
Guangxi	345.85	525.10	179.25	< 0.001
Ningxia	242.34	414.97	172.63	< 0.001
Yunnan	380.21	541.96	161.75	0.013
Chongqing	37.16	171.54	134.38	< 0.001
Shanghai	30.53	103.79	73.26	< 0.001
Tianjin	46.88	68.05	21.17	0.001
Inner Mongolia	683.44	702.89	19.45	0.729
Beijing	47.47	61.42	13.95	0.012
Liaoning	584.99	472.08	-112.91	0.145
Hainan	379.91	244.15	-135.76	0.048
Jilin	427.55	266.78	-160.77	< 0.001
Heilongjiang	652.25	426.68	-225.57	< 0.001

^a^Annual average during 2000–2021. ^*^Difference = value in 2022 - annual average value during 2000−2021. *CEDD* cumulative excessive degree-day

**Table 3:** Differences in the person-days of heatwave exposure for the population aged 0–64 years and ≥ 65 years between 2022 and the annual average during 2000–2021 in China

Province/Municipality	Population aged 0–64 years (million, person-days)				Population aged ≥ 65 years (million, person-days)			
	2000–2021^a^	2022	Difference^*^	*P*-value	2000–2021^a^	2022	Difference^*^	*P*-value
Sichuan	1505.49	3277.70	1772.21	< 0.001	192.57	668.09	475.52	< 0.001
Henan	2153.06	4191.20	2038.14	< 0.001	213.11	653.42	440.30	< 0.001
Jiangsu	1664.34	3322.33	1657.99	< 0.001	212.72	642.12	429.39	< 0.001
Hunan	1452.84	3063.95	1611.10	< 0.001	166.82	532.76	365.94	< 0.001
Zhejiang	1174.93	2855.96	1681.03	< 0.001	137.03	436.87	299.83	< 0.001
Shandong	2190.34	3088.02	897.68	< 0.001	258.83	550.64	291.80	< 0.001
Anhui	1357.25	2480.75	1123.50	< 0.001	155.34	438.08	282.74	< 0.001
Hubei	1309.79	2374.25	1064.45	< 0.001	137.66	405.48	267.82	< 0.001
Jiangxi	1040.12	2188.26	1148.14	< 0.001	95.37	295.18	199.81	< 0.001
Guangdong	2310.82	3902.44	1591.61	< 0.001	181.09	366.30	185.20	< 0.001
Hebei	1525.11	2011.64	486.53	< 0.001	151.00	325.38	174.39	< 0.001
Shanghai	474.08	1160.93	686.85	< 0.001	65.00	225.75	160.75	< 0.001
Chongqing	565.57	1107.91	542.34	< 0.001	73.45	228.15	154.70	< 0.001
Fujian	832.11	1663.19	831.07	< 0.001	77.87	207.62	129.75	< 0.001
Shaanxi	820.12	1273.12	453.00	< 0.001	78.14	195.70	117.55	< 0.001
Guizhou	669.85	1380.04	710.20	< 0.001	63.83	180.32	116.50	< 0.001
Shanxi	792.06	1081.31	289.25	< 0.001	71.67	160.17	88.50	< 0.001
Gansu	499.89	883.25	383.36	< 0.001	42.94	127.12	84.17	< 0.001
Guangxi	954.31	1306.26	351.95	< 0.001	100.65	181.46	80.81	< 0.001
Beijing	413.45	761.72	348.27	< 0.001	45.96	116.86	70.90	< 0.001
Liaoning	873.51	750.46	-123.05	0.138	110.96	158.26	47.30	0.002
Tianjin	271.81	418.28	146.46	< 0.001	29.85	72.39	42.53	< 0.001
Yunnan	694.58	867.39	172.80	0.059	62.25	104.43	42.18	< 0.001
Heilongjiang	753.00	612.89	-140.11	0.012	74.07	113.40	39.33	< 0.001
Xinjiang	476.03	858.47	382.44	< 0.001	33.21	72.21	39.00	< 0.001
Inner Mongolia	520.30	543.52	23.22	0.471	46.36	81.59	35.23	< 0.001
Qinghai	108.25	257.84	149.59	< 0.001	7.40	24.50	17.10	< 0.001
Ningxia	139.13	230.26	91.13	< 0.001	9.66	24.51	14.84	< 0.001
Xizang	61.66	184.20	122.54	< 0.001	3.63	11.08	7.45	< 0.001
Hainan	192.59	219.60	27.01	0.383	18.23	25.57	7.34	0.037
Jilin	534.37	321.15	-213.22	< 0.001	54.75	59.39	4.64	0.459

^a^Annual average during 2000–2021. ^*^Difference = value in 2022 - annual average value during 2000−2021

### Mortality burden attributable to the heatwaves

From 2000 to 2022, the total number of deaths attributable to heatwaves amounted to 854,683 cases. The distribution of heatwave-related AN for each individual heatwave across all cities is presented in Additional file 1: Fig. S3, with a median of 9 deaths per heatwave. During this period, heatwave-related AN and AF of total deaths exhibited an increasing trend, with rates of 1283 cases and 0.20‰ per year, respectively (both *P* < 0.05; Additional file 1: Figs. S4, S5). In 2022, heatwaves were estimated to be responsible for 62,961 (95% CI 54,945–70,413) deaths across China. Among these deaths, 40,567 (95% CI 35,313–45,404) were attributed to cardiovascular diseases, 35,876 (95% CI 31,035–41,005) involved females, and 56,208 (95% CI 49,023–62,864) occurred among individuals over 65 years of age. The AF of heatwave-related deaths was 18.11‰ (95% CI 15.80–20.25), with considerable variation across subgroups, ranging from 7.69‰ (95% CI 4.18–10.98) in people aged 0–64 years to 24.33‰ (95% CI 21.18–27.23) for cardiovascular diseases. Compared to the annual average during 2000–2021, we observed significant increases in the AN and AF of heatwave-related deaths in 2022. For instance, the rate of increase in the AF of non-accidental mortality was 64.43% (95% CI 38.10–93.78), with varying rates ranging from 58.77% (95% CI 33.38–90.76) in cardiovascular diseases to 76.19% (95% CI 19.42–157.43) in respiratory diseases (Table 4).

**Table 4:** Comparison of the attributable number (AN) and attributable fraction (AF) of deaths attributable to heatwaves in 2022 with the average during 2000–2021 in China

Item	AN				AF			
	2000–2021 [*n* (95% CI)] ^a^	2022 [*n* (95% CI)]	Percentage change (%)	*P*-value	2000–2021 [‰ (95% CI)] ^a^	2022 [‰ (95% CI)]	Percentage change (%)	*P*-value
Non-accidental mortality	35,987 (31,252–40,471)	62,961 (54,945–70,413)	74.95 (46.94–106.18)	< 0.001	11.01 (9.56–12.38)	18.11 (15.80–20.25)	64.43 (38.10–93.78)	< 0.001
Cause-specific								
Cardiovascular diseases	23,402 (20,259–26,347)	40,567 (35,313–45,404)	73.35 (45.62–108.28)	< 0.001	15.32 (13.27–17.25)	24.33 (21.18–27.23)	58.77 (33.38–90.76)	< 0.001
Respiratory diseases	4745 (3439–5954)	7721 (5609–9630)	62.71 (10.28–137.73)	0.013	11.63 (8.43–14.60)	20.50 (14.89–25.57)	76.19 (19.42–157.43)	0.004
Other diseases	7395 (4708–10,052)	13,818 (9319–18,249)	86.85 (17.02–212.74)	0.015	5.55 (3.53–7.54)	9.64 (6.50–12.73)	73.79 (8.84–190.89)	0.030
Sex								
Male	15,089 (12,136–18,161)	26,317 (21,539–31,394)	74.41 (32.43–129.80)	< 0.001	8.13 (6.54–9.78)	13.25 (10.84–15.80)	63.00 (23.77–114.77)	< 0.001
Female	20,512 (17,742–23,531)	35,876 (31,035–41,005)	74.90 (74.56–75.30)	< 0.001	14.54 (12.58–16.68)	24.08 (20.83–27.52)	65.57 (65.24–65.94)	< 0.001
Age (years)								
0–64	4196 (2153–6130)	5763 (3132–8231)	37.36 (-29.79 to 194.63)	0.342	4.69 (2.41–6.85)	7.69 (4.18–10.98)	63.99 (-16.17 to 251.76)	0.147
≥ 65	29,951 (25,996–33,730)	56,208 (49,023–62,864)	87.67 (56.15–124.81)	< 0.001	13.15 (11.41–14.81)	21.64 (18.88–24.20)	64.56 (36.93–97.13)	< 0.001

^a^Annual average during 2000–2021. *AN* attributable number, *AF* attributable fraction, *CI* confidence interval

We also found significant spatial variations in the heatwave-related mortality burden in China, with a higher burden observed in Central-Eastern China. Specifically, in 2022, the top 3 provinces with the highest AN attributed to heatwaves were Shandong (6425, 95% CI 5615–7242), Henan (6094, 95% CI 4377–7759), and Jiangsu (5753, 95% CI 5034–6458). The top 3 provinces with the highest AF were Zhejiang (33.19‰, 95% CI 28.93–37.55), Anhui (31.59‰, 95% CI 27.63–35.72), and Jiangsu (30.40‰, 95% CI 26.61–34.13). Moreover, compared with the average level during 2000–2021, we observed greater increases in the number of deaths and AF attributable to heatwaves in Central-Eastern China and the Qinghai-Xizang Plateau in 2022 (Table 5 and Additional file 1: Tables S8, S9). For example, the 3 provincial-level administrative regions with the highest percentage change in AF were Xizang, Sichuan, and Chongqing. The cause-specific, age, and gender subgroups also exhibited similar spatial heterogeneity, with details shown in Additional file 1: Figs. S6, S7.

**Table 5:** Comparison in the attributable number (AN) of deaths and attributable fraction (AF) to heatwaves in 2022 and the annual average during 2000 – 2021

Province/Municipality	AN				AF			
	2000–2021 (95% CI) ^a^	2022 (95% CI)	Percentage change (%)	*P*-value	2000–2021 [‰ (95% CI)] ^a^	2022 [‰ (95% CI)]	Percentage change (%)	*P*-value
Xizang	48 (25–72)	121 (62–180)	151.14 (9.09–458.66)	0.025	6.04 (3.08–8.99)	15.69 (8.00–23.39)	159.77 (12.84–477.87)	0.021
Sichuan	1505 (767–2241)	3620 (1840–5441)	140.54 (6.86–431.86)	0.033	6.19 (3.15–9.22)	14.46 (7.35–21.74)	133.64 (3.80–416.61)	0.037
Chongqing	604 (308–900)	1351 (679–2018)	123.69 (3.65–382.45)	0.045	6.52 (3.32–9.70)	14.35 (7.21–21.42)	120.22 (2.04–374.95)	0.048
Guizhou	671 (498–852)	1376 (1030–1730)	104.93 (39.75–191.09)	< 0.001	8.58 (6.37–10.90)	18.54 (13.87–23.31)	115.93 (47.25–206.71)	< 0.001
Zhejiang	1771 (1546–2006)	3941 (3435–4460)	122.57 (85.38–166.45)	< 0.001	15.40 (13.45–17.45)	33.19 (28.93–37.55)	115.49 (79.48–157.97)	< 0.001
Hunan	1726 (1282–2191)	3933 (2945–4991)	127.89 (56.51–230.75)	< 0.001	10.02 (7.44–12.72)	21.47 (16.07–27.24)	114.23 (47.13–210.92)	< 0.001
Qinghai	59 (30–88)	130 (65–195)	120.56 (0.70–381.80)	0.049	5.88 (2.98–8.75)	12.37 (6.17–18.54)	110.37 (-3.95 to 359.53)	0.062
Jiangxi	952 (707–1212)	2057 (1534–2604)	116.10 (47.17–220.46)	< 0.001	10.29 (7.63–13.10)	20.51 (15.30–25.97)	99.45 (35.83–195.76)	< 0.001
Jiangsu	3009 (2637–3392)	5753 (5034–6458)	91.16 (62.04–126.86)	< 0.001	15.33 (13.43–17.28)	30.40 (26.61–34.13)	98.32 (68.11–135.35)	< 0.001
Henan	2948 (2118–3739)	6094 (4377–7759)	106.70 (39.00–202.99)	0.001	12.34 (8.87–15.65)	24.38 (17.51–31.04)	97.53 (32.83–189.55)	0.001
Hubei	1590 (1182–2025)	3371 (2493–4264)	112.00 (44.75–207.93)	< 0.001	10.36 (7.70–13.19)	20.41 (15.09–25.81)	97.02 (34.52–186.16)	0.001
Anhui	2521 (2204–2841)	4977 (4353–5627)	97.43 (67.19–139.71)	< 0.001	16.43 (14.37–18.51)	31.59 (27.63–35.72)	92.25 (62.81–133.42)	< 0.001
Fujian	703 (522–894)	1262 (924–1617)	79.46 (22.18–169.57)	0.005	10.00 (7.42–12.71)	19.07 (13.96–24.44)	90.74 (29.86–186.52)	0.002
Shanghai	735 (642–829)	1660 (1454–1868)	125.75 (87.49–169.53)	< 0.001	14.59 (12.74–16.44)	27.77 (24.32–31.24)	90.36 (58.09–127.27)	< 0.001
Gansu	360 (184–536)	681 (347–1015)	89.07 (-11.23 to 295.33)	0.095	6.22 (3.18–9.27)	11.27 (5.74–16.79)	81.02 (-15.01 to 278.51)	0.117
Xinjiang	267 (136–398)	420 (212–623)	57.43 (-23.63 to 236.94)	0.218	6.65 (3.39–9.92)	10.45 (5.28–15.51)	57.21 (-23.74 to 236.47)	0.219
Shaanxi	641 (326–955)	1085 (556–1625)	69.40 (-21.57 to 266.80)	0.159	7.07 (3.60–10.54)	10.98 (5.62–16.44)	55.21 (-28.13 to 236.09)	0.233
Ningxia	75 (38–112)	106 (54–159)	41.96 (-35.96 to 219.04)	0.335	6.89 (3.49–10.29)	10.14 (5.18–15.15)	47.19 (-33.60 to 230.80)	0.290
Guangdong	1508 (1118–1920)	2016 (1510–2556)	33.69 (-9.52 to 95.83)	0.131	9.36 (6.94–11.92)	13.71 (10.27–17.38)	46.52 (-0.83 to 114.63)	0.049
Shandong	4107 (3605–4613)	6425 (5615–7242)	56.43 (31.96–86.83)	< 0.001	16.75 (14.71–18.82)	23.10 (20.19–26.04)	37.89 (16.32–64.68)	< 0.001
Beijing	395 (283–503)	662 (480–840)	67.52 (14.49–146.34)	0.013	11.08 (7.93–14.09)	15.23 (11.04–19.32)	37.53 (-6.01 to 102.23)	0.114
Guangxi	950 (706–1203)	1322 (984–1676)	39.17 (-4.74 to 103.77)	0.086	9.33 (6.93–11.82)	12.61 (9.39–15.99)	35.21 (-7.45 to 97.97)	0.116
Shanxi	973 (699–1235)	1386 (1000–1759)	42.38 (-4.67 to 111.81)	0.082	11.85 (8.51–15.04)	15.50 (11.18–19.68)	30.78 (-12.44 to 94.55)	0.181
Hebei	2362 (1701–2994)	3594 (2591–4544)	52.20 (2.95–126.17)	0.039	11.58 (8.34–14.68)	14.85 (10.71–18.77)	28.26 (-13.24 to 90.59)	0.211
Tianjin	375 (270–477)	557 (395–706)	48.38 (-0.73 to 114.19)	0.056	11.23 (8.09–14.28)	14.25 (10.12–18.08)	26.93 (-15.08 to 83.24)	0.239
Yunnan	494 (251–736)	631 (329–942)	27.88 (-37.81 to 174.49)	0.490	4.78 (2.43–7.12)	5.67 (2.95–8.46)	18.70 (-42.28 to 154.77)	0.628
Hainan	132 (98–168)	142 (105–179)	7.15 (-27.26 to 54.59)	0.715	9.70 (7.22–12.34)	10.02 (7.40–12.63)	3.33 (-29.86 to 49.08)	0.863
Inner Mongolia	758 (543–962)	847 (607–1072)	11.85 (-26.63 to 65.69)	0.574	11.09 (7.95–14.09)	11.31 (8.10–14.31)	1.97 (-33.11 to 51.06)	0.921
Liaoning	1668 (1197–2119)	1627 (1155–2073)	-2.49 (-31.80 to 46.10)	0.900	11.10 (7.96–14.09)	9.66 (6.86–12.30)	-12.99 (-39.14 to 30.38)	0.490
Heilongjiang	1250 (899–1584)	1214 (877–1549)	-2.86 (-35.67 to 42.67)	0.883	10.65 (7.66–13.50)	8.83 (6.39–11.27)	-17.06 (-45.07 to 21.82)	0.349
Jilin	830 (596–1055)	600 (428–764)	-27.76 (-50.87 to 7.53)	0.112	10.69 (7.67–13.58)	6.67 (4.76–8.49)	-37.62 (-57.58 to -7.15)	0.024

^a^Annual average during 2000–2021. *AN* attributable number, *AF* attributable fraction, *CI* confidence interval

### Sensitivity analyses

Sensitivity analyses demonstrated the robustness of our findings by changing the maximum lag days and *df* of the time variable and adjusting for ambient air pollutants. Mortality risk increased with the percentile of the threshold of the heatwave definition; however, the corresponding AN and AF showed fluctuations. The AN and AF calculated based on trend-estimated mortality rate were similar to those calculated assuming a constant mortality rate after 2019. Heatwave-related excess risk was slightly higher in 2006–2011 than in 2013–2017. Compared to heatwaves defined by maximum temperature, those defined by minimum temperature and heat index are associated with both lower risks and mortality burdens (Additional file 1: Table S10).

## Discussion

This nationwide study provides a comprehensive analysis of the characteristics of heatwaves and their associated mortality burden in China over the last 20 years. The results showed that the mortality burden attributable to heatwaves had increased significantly in the past 2 decades, reaching a peak of over 60 thousand deaths in 2022. The mortality burden was particularly pronounced for cardiovascular diseases, Central-Eastern China, females, and the elderly. These results imply the mortality burden of heatwaves may continuously increase in the future due to the combination of ongoing climate change and rapid aging in China.

In this study, we employed the CEDD indicator, which simultaneously considers the frequency and intensity of heatwaves, to describe the spatiotemporal changes in heatwaves in China over the past 2 decades. We observed a significant increasing trend in CEDD from 2000 to 2022, with the highest levels occurring in 2022. Spatially, the increase in CEDD was greater in Central-Eastern China and on the Qinghai-Xizang Plateau. Furthermore, we employed the person-day metric to quantify heatwave exposure, which is defined as the product of the number of days exceeding the heatwave threshold and the size of the affected population. By combining the heatwave duration with the number of people affected, the person-day metric provides a more comprehensive reflection of the overall population-level exposure to heatwaves. Meanwhile, the metric of person-day serves as a standardized quantitative indicator, facilitating comparisons of heatwave exposure levels across different regions and time periods. It also helps distinguish the relative contributions of population size and temperature increase to the rise in heatwave exposure. In recent years, studies using the person-day to define heatwave exposures have been increasing [51, 52]. For public health practice, the person-day metric can directly reflect the severity of a heatwave and be used to assess the potential impacts of heatwaves on public health, thereby supporting evidence-based resource allocation and response strategies by relevant authorities. In our study, the person-days for adults aged 65 years and above, and those in the central region were particularly high in 2022, highlighting the vulnerability of the population to the unprecedented heatwaves.

Consistent with previous studies [17, 41], the findings indicate that heatwave was associated with an 8.4% increase in non-accidental mortality across China. Based on this association, we estimated that 62,961 deaths were attributed to the 2022 heatwaves, an increase of 74.95% compared to the 2000–2021 annual average, and suggesting it may rank among the deadliest heatwaves of the 21st century. This finding aligns with what has been observed in Europe [29], where more than 61 thousand heat-related deaths were reported during the summer of 2022, approaching the record-breaking excess mortality observed in the 2003 heatwaves, despite the implementation of heat prevention plans since then [27]. Furthermore, a global study also found that the heat-related excess deaths in East Asia have shown a fluctuating upward trend over the past 2 decades [37]. These results suggest that extensive and persistent heatwaves result in a substantial mortality burden, even in developed countries with better adaptation resources, indicating that current adaptation strategies and measures might not be effective enough in protecting the health of vulnerable populations in developed and developing countries.

In terms of demographic characteristics, females exhibited higher vulnerability to heatwaves than males, including both heatwave-related risks and the attributable burden. This disparity may be related to the variation in exposure patterns, lifestyles, social roles, thermoregulatory mechanisms, and physiological structure [53, 54]. For instance, females typically demonstrated higher core body temperatures, skin temperatures, and heart rates, which may have reduced their heat tolerance [54]. The findings underscore the heightened vulnerability of the elderly population during heatwaves, particularly in 2022, which was consistent with previous studies [41, 55, 56]. First, due to age-related declines in thermoregulatory capacity and disruptions in homeostasis, the elderly population struggled to cope with heatwaves [57, 58], leading to greater associated risks and burdens. Second, some elderly individuals experienced insufficient social support and self-care, which may have further heightened their vulnerability [59]. Third, the elderly were more likely to suffer from cardiovascular and respiratory diseases, and heatwaves could impose additional physiological strain, such as increased blood viscosity, cholesterol levels, red blood cell count, platelet count, and allergen proliferation [60–62]. The findings also revealed that individuals with cardiopulmonary diseases were more susceptible to heatwaves, consistent with previous studies [63, 64]. Finally, population aging may be a key driver of the increased heatwave-related mortality burden among the elderly in 2022. The aging of the Chinese population is accelerating, with the proportion of individuals over 65 years of age reaching 190 million (13.5% of the total population) in 2020 [65]. It is projected that the proportion of the elderly will continuously increase in the coming decades in China, exceeding 30% by 2050 [66]. Notably, beyond females and the elderly, some other populations are also required attention due to their heightened vulnerability to heatwaves, such as construction laborers, sanitation workers, and military personnel. A previous study found that a 1 °F (0.55 °C) increase in mean temperature during May-September was associated with a 1.16-fold higher outpatient rate of heat stress illness among United States soldiers [67]. Therefore, targeted interventions were essential to mitigate the adverse health effects of heatwaves such as developing real-time monitoring mechanisms for heat-related health risks based on artificial intelligence (AI) technology, establishing dedicated “heatwave clinics”, enhancing social support for those vulnerable groups, and improving their living environments.

Furthermore, we observed significant spatial variations in heatwave-related mortality, with a higher mortality burden in Central-Eastern China, which has also been reported in a previous study [31]. On the one hand, Central-Eastern China is characterized by subtropical and temperate monsoon climates, where summer heatwaves exhibit greater intensity and longer duration. Meanwhile, these eastern areas have high urbanization rates, and the urban heat island (UHI) may further amplify heatwave effects [68]. On the other hand, the eastern region is among the most densely populated areas in the country, with a notably aging population, which increases heatwave-related risks and burdens. Additionally, we observed a greater increase in mortality burdens due to heatwaves in the Qinghai-Xizang Plateau, particularly in Xizang, in 2022 compared with 2000–2021. This phenomenon may be driven by multiple factors. (1) Although the Qinghai-Xizang Plateau has a relatively low population size and density, its high-altitude location is associated with inherently low baseline temperatures. Climate change-induced temperature rises have therefore led to a marked increase in heatwave exposure, which in turn drives significant changes in heatwave-attributable mortality burdens. (2) The economic and healthcare levels in this region are relatively underdeveloped, rendering residents more vulnerable to heatwaves. These findings provide critical scientific evidence for formulating climate change adaptation measures and policies from a public health perspective. Specifically, there are 2 key implications: 1) heatwave-related health risks and burdens in Central-Eastern China have likely emerged as a pressing regional public health concern, indicating the need to establish clear response prioritization. Intervention and adaptation measures such as optimizing urban form, mitigating the urban heat island effect, and implementing heat protection for workers (especially outdoor workers) can be adopted; 2) heatwave health risk warnings and interventions should be tailored to local conditions. Particularly in the context of global warming, the potential risks of substantial increases in heatwave-related health burdens in the Qinghai-Xizang Plateau may increase significantly. Therefore, it is advisable to lower the warning threshold for heatwave health risks while strengthening public awareness campaigns regarding the health hazards of high temperatures.

Although the unprecedented heatwaves in 2022 have caused a serious adverse health burden, global warming may continue to increase in the coming years, even if strict mitigation actions are adopted immediately [2]. Recently, United Nations chief Antonio Guterres stated, “The era of global warming has ended; the era of global boiling has arrived” [69]. Moreover, the China Meteorological Administration reported that the average temperature in July 2023 across China was 23.0 °C, which was almost the same as that (23.2 °C) in July 2022. Therefore, several adaptation options are suggested to reduce the adverse health impacts of future increasing heatwaves. (1) Rigorous strategies and measures across multiple departments and systems are urgently needed to mitigate greenhouse gas emissions over the long term [70]. (2) A national health adaptation plan for climate change should be developed and implemented across government departments, incorporating key measures such as national early heat warning systems [71]. (3) More effective risk communications are needed to raise public awareness of heatwaves and their health impacts [72]. (4) Targeted adaptation measures should be implemented to protect vulnerable populations such as the elderly, women, and people with chronic diseases. For example, cooling centers can be set up in the community to provide residents with a safe place to handle the heatwaves [73].

This study had several limitations. (1) Due to restrictions on accessing complete death records, we utilized mortality data from 364 locations during the period 2006–2017 as a sample to estimate the heatwave-mortality association. These associations were then extrapolated to assess the national heatwave-related burden from 2000–2022. Therefore, potential inadequacies in sample representativeness (e.g., sparse samples in Western regions) and potential deficiencies in the extrapolation process may introduce uncertainties in assessing heatwave-related mortality burdens. Additionally, the unavailability of more detailed disease classification data prevented a precise assessment of heatwave-related burdens for specific disease subtypes (e.g., stroke, asthma) and external causes of death (e.g., traffic injuries, falls). (2) The person-days of heatwave exposure were only evaluated in subgroups stratified by age and city development level. Due to data accessibility limitations, we were unable to explore the spatial heterogeneity of heatwave exposure across more spatial dimensions (e.g., urban vs. rural areas). Third, the assumption of the same annual mortality rate from 2020 to 2022 may also lead to uncertainty in the mortality burden estimates. However, the results of sensitivity analyses using mortality rate based on trend estimation indicated the robustness of our findings. Fourth, variations in the number of study locations between 2006–2011 and 2013–2017 were observed due to the expansion of mortality surveillance sites after 2013 [33]. However, sensitivity analyses indicated no significant changes in the heatwave-mortality relationship between these 2 periods. Finally, although ERA5 has demonstrated good performance in validation against meteorological station data, its spatial resolution (0.25° × 0.25°) may limit the identification of urban microclimates and lead to misclassification. Additionally, as this is an ecological study, caution is needed regarding potential ecological fallacies.

## Conclusions

This nationwide analysis indicated the frequency and intensity of heatwaves increased in the past two decades in China, and more intensive and frequent heatwaves in 2022 were linked to a substantial mortality burden, with significant population and regional heterogeneity. Given the dual drivers of global warming and rapid aging in China, it is imperative to adopt more assertive adaptation strategies to protect human health from heatwaves.

## Supplementary Information

**Additional file 1**.**Table S1** Akaike Information Criterion (AIC) values in the model fitting using various heatwave definitions.** Table S2** The characteristics of daily non-accidental mortality and environmental variables in 364 locations during the warm seasons of 2006–2017. **Table S3 **The average numbers of daily non-accidental mortality during the warm seasons from 2006 to 2017 by heatwave and non-heatwave days (mean ± standard deviation). **Table S4** Comparison of the characteristics of heat waves in warm season in 2022 with that during 2000–2021 in China. **Table S5** Spatial distribution of the difference between the average daily maximum temperature during the heatwave in 2022 and the average during 2000–2021 at the province level in China. **Table S6** Spatial distribution of the difference between the number of heatwave days in 2022 and the annual average during 2000–2021 at the province level in China. **Table S7** The population exposure to heatwave in cities with different development levels (million person-days). **Table S8** Percentage change [% (95% CI)] in the attributable number (AN) of deaths from heatwaves in 2022 compared with the average during 2000−2021, by province, causes of death, sex, and age. **Table S9** Percentage change [% (95% CI)] in the AF of deaths attributable to heatwaves in 2022 compared to the average during 2000−2021, by province, causes of death, sex, and age. **Table S10** The sensitivity analyses of the excess risk (ER), AN, and AF. **Fig. S1** The lag patterns in the effects of various heatwave definitions on non-accidental mortality. **Fig. S2** The characteristics of heatwaves and average daily maximum temperature in 2022 compared with that during 2000–2021 in China. **Fig. S3** The frequency distribution of attributable deaths per heatwave event in each city of China during 2000–2022. **Fig. S4** The trend of AN of deaths related to heatwaves in China, 2000–2022. **Fig. S5** The trend of AF of death related to heatwaves in China, 2000–2022. **Fig. S6** The comparison of Comparison of the AF of death related to heatwaves at provincial level in 2022 with that during 2000–2021 by sex, age, and cause of death. **Fig. S7** The comparison of the AN of death related to heatwaves at the provincial level in 2022 with that during 2000–2021 by sex, age, and cause of death.

## Data Availability

Due to restrictions imposed by confidentiality agreements and relevant authority regulations, mortality data can be obtained from the corresponding authors upon reasonable request; other data (including geographic data, meteorological data, and air pollution data) are available directly at https://github.com/Hzeros/heatwave2022. The summary statistics and descriptive tables in this study are all included in the Supplementary Information.

## Abbreviations

AIC: Akaike information criterion
AN: Attributable number
AF: Attributable fraction
CEDD: Cumulative excessive degree-day
CI: Confidence interval
DLNM: Distributed lag nonlinear model
ERA5: The fifth-generation European Centre for Medium-Range Weather Forecasts atmospheric reanalysis
RMSE: Root mean square error
