# Supplementary material for: National mortality burden attributable to the unprecedented heatwave in 2022 in China
Source: Mil Med Res. 2025 Dec 15;12:92. doi: 10.1186/s40779-025-00676-2 (PMC12703895; doi:10.1186/s40779-025-00676-2)
Supplement: Supplementary file 1 — Additional file 1.Table S1 Akaike Information Criterion (AIC) values in the model fitting using various heatwave definitions. Table S2 The characteristics of daily non-accidental mortality and environmental variables in 364 locations during the warm seasons of 2006–2017. Table S3 The average numbers of daily non-accidental mortality during the warm seasons from 2006 to 2017 by heatwave and non-heatwave days (mean ± standard deviation). Table S4 Comparison of the characteristics of heat waves in warm season in 2022 with that during 2000–2021 in China. Table S5 Spatial distribution of the difference between the average daily maximum temperature during the heatwave in 2022 and the average during 2000–2021 at the province level in China. Table S6 Spatial distribution of the difference between the number of heatwave days in 2022 and the annual average during 2000–2021 at the province level in China. Table S7 The population exposure to heatwave in cities with different development levels (million person-days). Table S8 Percentage change [% (95% CI)] in the attributable number (AN) of deaths from heatwaves in 2022 compared with the average during 2000−2021, by province, causes of death, sex, and age. Table S9 Percentage change [% (95% CI)] in the AF of deaths attributable to heatwaves in 2022 compared to the average during 2000−2021, by province, causes of death, sex, and age. Table S10 The sensitivity analyses of the excess risk (ER), AN, and AF. Fig. S1 The lag patterns in the effects of various heatwave definitions on non-accidental mortality. Fig. S2 The characteristics of heatwaves and average daily maximum temperature in 2022 compared with that during 2000–2021 in China. Fig. S3 The frequency distribution of attributable deaths per heatwave event in each city of China during 2000–2022. Fig. S4 The trend of AN of deaths related to heatwaves in China, 2000–2022. Fig. S5 The trend of AF of death related to heatwaves in China, 2000–2022. Fig. S6 The comparison of Compari [file 40779_2025_676_MOESM1_ESM.pdf]

Table S1 Akaike information criterion (AIC) values in the model fitting using various heatwave definitions

Heatwave	Definition of heatwave	AIC
HW850.2	Daily maximum temperature ≥ 85.0th percentile for 2 or more consecutive days	4615.42
HW850.3	Daily maximum temperature ≥ 85.0th percentile for 3 or more consecutive days	4615.63
HW850.4	Daily maximum temperature ≥ 85.0th percentile for 4 or more consecutive days	4615.90
HW850.5	Daily maximum temperature ≥ 85.0th percentile for 5 or more consecutive days	4615.75
HW875.2	Daily maximum temperature ≥ 87.5th percentile for 2 or more consecutive days	4614.70
HW875.3	Daily maximum temperature ≥ 87.5th percentile for 3 or more consecutive days	4615.14
HW875.4	Daily maximum temperature ≥ 87.5th percentile for 4 or more consecutive days	4615.32
HW875.5	Daily maximum temperature ≥ 87.5th percentile for 5 or more consecutive days	4615.20
HW900.2	Daily maximum temperature ≥ 90.0th percentile for 2 or more consecutive days	4614.83
HW900.3	Daily maximum temperature ≥ 90.0th percentile for 3 or more consecutive days	4614.62
HW900.4	Daily maximum temperature ≥ 90.0th percentile for 4 or more consecutive days	4614.75
HW900.5	Daily maximum temperature ≥ 90.0th percentile for 5 or more consecutive days	4615.33
HW925.2	Daily maximum temperature ≥ 92.5th percentile for 2 or more consecutive days	4614.12
HW925.3	Daily maximum temperature ≥ 92.5th percentile for 3 or more consecutive days	4614.14
HW925.4	Daily maximum temperature ≥ 92.5th percentile for 4 or more consecutive days	4614.56
HW925.5	Daily maximum temperature ≥ 92.5th percentile for 5 or more consecutive days	4614.89
HW950.2	Daily maximum temperature ≥ 95.0th percentile for 2 or more consecutive days	4614.19
HW950.3	Daily maximum temperature ≥ 95.0th percentile for 3 or more consecutive days	4614.21
HW950.4	Daily maximum temperature ≥ 95.0th percentile for 4 or more consecutive days	4614.86
HW975.2	Daily maximum temperature ≥ 97.5th percentile for 2 or more consecutive days	4614.19
HW975.3	Daily maximum temperature ≥ 97.5th percentile for 3 or more consecutive days	4614.62

Table S2 The characteristics of daily non-accidental mortality and environmental variables in 364 locations during the warm seasons of 2006 – 2017

Item	Mean	SD	Minimum value	P_{25}	P_{50}	P_{75}	Maximum value
Non-accidental mortality	8	5	0	4	6	10	151
Cause-specific							
Cardiovascular diseases	3	3	0	1	2	4	69
Respiratory diseases	1	1	0	0	1	2	35
Other diseases	3	3	0	1	3	5	59
Sex							
Male	4	3	0	2	4	6	92
Female	3	3	0	1	3	4	61
Age (years)							
0 − 64	2	2	0	1	2	3	40
≥ 65	6	4	0	2	5	8	127
Meteorological variable							
Daily maximum temperature (℃)	27.9	5.1	-2.4	24.8	28.3	31.5	43.6
Humidity (%)	76.1	12.8	8.9	70.2	78.7	85.1	99.6
Air pollutant							
$PM_{2.5}$ (μg/m^3)	32.9	16.5	4.6	21	28.7	40.6	371.2
O_3 (μg/m^3)	99.8	28.8	6.9	78.1	96.6	117.1	330.6

SD standard deviation, P_{25} 25th percentile, P_{50} 50th percentile, P_{75} 75th percentile, $PM_{2.5}$ particulate matter with an aerodynamic diameter of ≤ 2.5 μm, O_3 ozone

Table S3 The average numbers of daily non-accidental mortality during the warm seasons from 2006 to 2017 by heatwave and non-heatwave days (mean ± standard deviation)

**Item**	**Heatwave day (*n* = 51,875)**	**Non-heatwave day (*n* = 292,222)**	***P*-value**
Non-accidental mortality	7.90 ± 5.73	7.43 ± 5.39	< 0.001
Cause-specific			
Cardiovascular diseases	3.17 ± 2.90	3.00 ± 2.78	< 0.001
Respiratory diseases	1.07 ± 1.40	1.02 ± 1.35	< 0.001
Other diseases	3.67 ± 3.12	3.41 ± 2.91	< 0.001
Sex			
Male	4.56 ± 3.57	4.35 ± 3.44	< 0.001
Female	3.34 ± 2.85	3.08 ± 2.63	< 0.001
Age (years)			
0 − 64	2.10 ± 1.95	2.03 ± 1.88	< 0.001
≥ 65	5.81 ± 4.53	5.39 ± 4.23	< 0.001

Table S4 Comparison of the characteristics of heat waves in warm season in 2022 with those during 2000 − 2021 in China

Characteristics	2000 − 2021				2022	Difference*	*P*-value
	**Annual maximum value**	**Annual minimum value**	**Annual median value**	**Annual average value**			
Total number of heatwaves (*n*)	2684	1642	2256	2189	2795	606	< 0.001
Average duration of heatwave (d)	4.45	3.50	3.85	3.95	5.09	1.14	< 0.001
Average maximum temperature (°C)	32.07	31.26	31.79	31.74	32.39	0.65	< 0.001
Cumulative excessive degree-day	20,470	8841	12,952	13,772	31,626	17,854	< 0.001
Person-days exposure to heatwave for the population aged 0 − 64 years (billion)	39.50	17.92	27.74	28.33	48.64	20.31	< 0.001
Person-days exposure to heatwave for the elderly aged ≥ 65 years (billion)	4.68	1.77	2.77	2.97	7.68	4.71	< 0.001

*Difference = value in 2022 − annual average value during 2000 − 2021

Table S5 Spatial distribution of the difference between the average daily maximum temperature during the heatwave in 2022 and the average during 2000 – 2021 in China

**Province/Municipality**	**2000 − 2021**				**2022**	**Difference***	***P*-value**
	**Annual maximum value**	**Annual minimum value**	**Annual median value**	**Annual average value**			
Henan	30.16	27.16	28.51	28.71	30.34	1.63	< 0.001
Chongqing	28.32	25.80	26.83	26.84	28.35	1.51	< 0.001
Anhui	29.95	28.18	29.27	29.15	30.64	1.49	< 0.001
Xinjiang	25.90	23.83	24.93	24.93	26.42	1.49	< 0.001
Hubei	29.31	27.25	28.46	28.45	29.93	1.48	< 0.001
Sichuan	26.26	24.19	24.98	25.02	26.45	1.43	< 0.001
Jiangsu	29.42	27.52	28.59	28.52	29.91	1.38	< 0.001
Qinghai	14.08	12.37	13.03	13.16	14.35	1.19	< 0.001
Xizang	11.90	10.33	11.53	11.25	12.41	1.15	< 0.001
Hunan	30.33	27.86	29.20	29.19	30.30	1.12	< 0.001
Gansu	22.44	21.05	21.60	21.6	22.73	1.12	< 0.001
Ningxia	25.8	23.89	24.59	24.62	25.72	1.09	< 0.001
Zhejiang	29.66	27.45	28.83	28.71	29.75	1.04	< 0.001
Jiangxi	31.35	29.19	30.46	30.41	31.4	0.98	< 0.001
Shanghai	28.33	26.38	27.50	27.45	28.38	0.93	< 0.001
Shaanxi	26.19	24.53	25.25	25.29	26.15	0.86	< 0.001
Shandong	28.99	26.35	27.64	27.68	28.53	0.85	< 0.001
Shanxi	25.64	23.48	24.74	24.61	25.38	0.77	< 0.001
Hebei	28.56	26.25	27.24	27.32	28.06	0.75	< 0.001
Tianjin	29.77	27.32	28.36	28.52	29.21	0.69	< 0.001
Guizhou	27.25	25.22	26.08	26.08	26.72	0.64	< 0.001
Beijing	28.12	26.05	27.00	27.05	27.65	0.60	< 0.001
Inner Mongolia	25.19	23.39	24.28	24.23	24.69	0.46	< 0.001
Fujian	30.29	28.69	29.32	29.37	29.55	0.18	0.084
Yunnan	25.27	23.03	24.36	24.16	24.19	0.03	0.821
Guangxi	31.32	29.39	30.2	30.19	30.1	-0.09	0.303
Guangdong	31.38	29.73	30.4	30.35	30.25	-0.10	0.211
Heilongjiang	23.74	21.87	22.59	22.58	22.46	-0.12	0.229
Hainan	31.38	29.71	30.17	30.34	30.21	-0.13	0.219
Liaoning	26.56	24.68	25.55	25.48	25.22	-0.26	0.043
Jilin	25.18	23.28	24.15	24.16	23.72	-0.44	< 0.001

*Difference = value in 2022 − annual average value during 2000 − 2021

Table S6 Spatial distribution of the difference between the number of heatwave days in 2022 and the annual average during 2000 − 2021 in China

Province/Municipality	2000 − 2021				2022	Difference*	*P*-value
	Annual maximum value	Annual minimum value	Annual median value	Annual average value			
Sichuan	969	235	378	440	1014	574	< 0.001
Hubei	738	185	434	427	870	443	< 0.001
Henan	748	78	448	449	865	416	< 0.001
Hunan	591	110	347	343	741	398	< 0.001
Anhui	701	210	421.5	405	773	368	< 0.001
Zhejiang	470	98	259.5	263	567	304	< 0.001
Jiangsu	614	170	298	310	613	303	< 0.001
Jiangxi	449	124	281	278	552	274	< 0.001
Xinjiang	764	249	526.5	542	810	268	< 0.001
Xizang	241	33	180.5	148	381	233	< 0.001
Guangdong	760	263	465.5	489	712	223	< 0.001
Gansu	516	142	278.5	299	522	223	< 0.001
Fujian	392	106	218.5	220	429	209	< 0.001
Guizhou	373	78	185.5	188	397	209	< 0.001
Qinghai	318	85	145	164	341	177	< 0.001
Shandong	695	91	384	403	561	158	< 0.001
Shaanxi	423	134	231	241	378	137	< 0.001
Guangxi	557	115	322.5	318	421	103	< 0.001
Shanxi	405	88	255.5	262	356	94	< 0.001
Hebei	401	118	247.5	254	338	84	< 0.001
Yunnan	594	38	277.5	256	337	81	0.014
Ningxia	183	58	115	117	175	58	< 0.001
Chongqing	46	6	20	22	49	27	< 0.001
Shanghai	50	8	21.5	22	43	21	< 0.001
Inner Mongolia	418	125	286	296	309	13	0.459
Hainan	1083	79	380	442	454	12	0.852
Beijing	39	5	22	22	31	9	< 0.001
Tianjin	41	6	23	23	29	6	0.003
Heilongjiang	457	95	291.5	286	249	-37	0.059
Liaoning	620	137	313	311	257	-54	0.064
Jilin	318	89	212.5	196	123	-73	< 0.001

*Difference = value in 2022 − annual average value during 2000 − 2021

Table S7 The population exposure to heatwave in cities with different development levels (million person-days)

**Group**	**High-development cities (*n* = 122)**	**Medium-development cities (*n* = 122)**	**Low-development cities (*n* = 123)**
0 − 64 years group			
2000 − 2021 (mean ± SD)	140.47 ± 32.56	68.18 ± 12.18	23.37 ± 4.00
2022	252.96	109.66	35.76
Difference*	112.48	41.48	12.39
P-value	< 0.001	< 0.001	< 0.001
≥ 65 years group			
2000 − 2021 (mean ± SD)	15.12 ± 5.47	7.01 ± 2.31	2.21 ± 0.78
2022	40.57	17.35	5.03
Difference*	25.45	10.34	2.82
P-value	< 0.001	< 0.001	< 0.001

*Difference = value in 2022 − annual average during 2000 − 2021. High-development cities: annual average GDP ≥ 12.50 million Yuan, medium-development cities: 5.53 million Yuan ≤ annual average GDP < 12.50 million Yuan, low-development cities: annual average GDP < 5.53 million Yuan. SD standard deviation

Table S8 Percentage change [% (95% CI)] in the attributable number (AN) of deaths from heatwaves in 2022 compared with the average during 2000 − 2021, causes of death, sex, and age

Parameter	Cause-specific			Sex		Age (years)	
	**Cardiovascular diseases**	**Respiratory diseases**	**Other diseases**	**Male**	**Female**	**0 − 64**	**≥ 65**
**National**	73.35 (45.62 – 108.28)	62.71 (10.28 – 137.73)	86.85 (17.02 – 212.74)	74.41 (32.43 – 129.80)	74.90 (74.56 – 75.30)	37.36 (-29.79 to 194.63)	87.67 (56.15 – 124.81)
**Provincial level**							
Xizang	145.29 (-33.90 to 668.48)	158.58 (-734.92 to 1661.07)	157.06 (-73.51 to 1157.18)	162.36 (-42.40 to 993.01)	139.03 (-19.13 to 705.38)	144.98 (-2476.05 to 1817.08)	115.50 (0.16 – 387.92)
Sichuan	141.12 (-31.85 to 774.57)	142.01 (-317.83 to 1700.45)	139.37 (-73.23 to 1226.92)	143.87 (-56.01 to 731.98)	136.43 (-31.31 to 690.42)	75.32 (-1071.37 to 1256.62)	156.11 (24.68 to 490.74)
Hunan	126.40 (54.42 – 235.82)	107.21 (-2.40 to 299.32)	137.66 (-789.16 to 2054.43)	127.68 (11.54 – 375.69)	128.32 (55.45 – 231.08)	76.26 (-917.18 to 1964.23)	142.84 (64.59 – 255.92)
Shanghai	133.55 (79.59 – 206.00)	88.39 (-1.83 to 223.69)	129.70 (55.79 – 230.84)	126.21 (66.81 – 199.54)	125.29 (79.73 – 179.28)	88.60 (22.19 – 195.15)	146.68 (109.60 – 190.09)
Chongqing	116.54 (-42.53 to 707.74)	116.46 (-870.57 to 1650.20)	133.76 (-69.37 to 825.09)	127.12 (-52.39 to 725.73)	119.03 (-34.80 to 568.25)	68.56 (-897.10 to 1526.43)	138.88 (-0.46 to 436.23)
Zhejiang	122.94 (66.29 – 191.59)	75.56 (-3.67 to 189.00)	136.64 (60.05 – 250.42)	126.02 (72.10 – 201.87)	118.39 (74.23 – 169.38)	97.16 (24.47 – 205.33)	126.79 (92.06 – 169.51)
Qinghai	130.62 (-33.78 to 707.03)	103.64 (-409.85 to 1132.69)	117.58 (-71.41 to 1114.94)	120.54 (-70.39 to 807.53)	120.48 (-29.71 to 580.90)	79.13 (-950.85 to 1768.31)	125.56 (-6.59 to 410.42)
Jiangxi	123.07 (53.06 – 235.08)	95.43 (-6.72 to 324.77)	116.09 (-620.94 to 1846.91)	116.94 (5.85 – 342.76)	115.09 (43.84 – 223.33)	71.85 (-1456.77 to 2097.76)	127.17 (54.89 – 240.58)
Hubei	108.96 (39.96 – 221.17)	105.96 (-4.83 to 318.28)	117.68 (-891.34 to 1603.67)	111.94 (4.17 – 298.78)	112.27 (36.73 – 213.73)	61.22 (-1683.45 to 1439.53)	125.10 (51.88 – 235.09)
Henan	114.89 (50.50 – 209.25)	62.96 (-231.54 to 900.49)	105.48 (-2830.59 to 2236.12)	103.79 (8.73 – 271.83)	110.05 (23.48 – 264.98)	62.98 (-325.24 to 1150.88)	127.05 (55.18 – 240.80)
Guizhou	101.28 (32.35 – 200.11)	90.79 (-0.67 to 289.22)	115.13 (-670.04 to 1320.15)	106.21 (7.67 – 309.35)	102.85 (35.38 – 189.85)	66.89 (-1523.51 to 1330.40)	112.07 (46.17 – 212.63)
Anhui	109.74 (63.37 – 173.28)	65.41 (-8.91 to 167.60)	93.05 (28.99 – 183.73)	97.94 (47.61 – 167.68)	96.72 (55.53 – 140.61)	44.02 (-5.15 to 118.68)	109.83 (78.39 – 148.45)

Parameter	Cause-specific			Sex		Age (years)	
	Cardiovascular diseases	Respiratory diseases	Other diseases	Male	Female	0 − 64	≥ 65
Jiangsu	83.37 (38.73 – 137.12)	81.58 (6.20 – 194.33)	100.61 (32.68 – 198.42)	95.96 (48.89 – 161.36)	85.66 (49.26 – 129.24)	44.50 (-8.34 to 126.96)	103.02 (72.41 – 140.12)
Gansu	100.47 (-48.98 to 607.28)	66.79 (-414.60 to 1286.63)	86.01 (-64.14 to 582.22)	84.16 (-66.93 to 549.84)	95.69 (-34.03 to 579.00)	32.20 (-198.26 to 727.96)	106.72 (-10.66 to 362.33)
Fujian	78.83 (19.69 – 172.01)	52.69 (-40.90 to 248.20)	86.70 (-586.69 to 1430.78)	79.73 (-11.63 to 247.17)	79.45 (19.43 – 178.51)	53.02 (-1078.21 to 1052.08)	86.51 (30.92 – 185.53)
Shaanxi	80.57 (-46.10 to 692.39)	30.95 (-610.62 to 708.71)	64.87 (-87.56 to 712.54)	69.01 (-70.23 to 606.93)	70.25 (-44.53 to 400.01)	23.19 (-732.25 to 731.33)	80.86 (-19.12 to 287.04)
Beijing	55.90 (6.75 – 128.68)	68.84 (-716.14 to 1079.99)	81.30 (-1614.70 to 2613.21)	65.84 (-7.41 to 197.65)	69.45 (-0.14 to 177.81)	37.42 (-872.80 to 945.37)	81.89 (26.64 – 175.38)
Xinjiang	64.07 (-44.93 to 467.95)	25.91 (-473.44 to 817.06)	61.24 (-85.52 to 723.07)	55.73 (-74.69 to 436.81)	60.02 (-50.45 to 399.93)	36.15 (-605.02 to 1131.68)	51.09 (-35.07 to 240.23)
Shandong	61.00 (20.57 – 117.01)	31.65 (-32.12 to 138.25)	57.18 (2.11 – 130.47)	60.24 (20.55 – 116.26)	51.60 (22.49 – 84.28)	27.29 (-20.48 to 100.59)	68.82 (42.85 – 98.37)
Hebei	58.66 (11.84 – 129.24)	16.22 (-257.35 to 687.31)	49.25 (-1084.38 to 1267.18)	55.87 (-15.32 to 198.84)	47.36 (-17.16 to 146.44)	21.21 (-474.08 to 826.65)	71.07 (17.58 – 163.28)
Tianjin	44.84 (-0.31 to 110.71)	32.96 (-665.54 to 1061.29)	56.61 (-1898.83 to 1798.64)	48.59 (-18.17 to 174.31)	47.70 (-12.78 to 159.87)	22.41 (-389.12 to 828.26)	63.08 (10.93 – 147.33)
Shanxi	48.71 (4.26 – 110.52)	23.83 (-404.93 to 704.20)	39.23 (-1526.19 to 1761.82)	42.18 (-22.71 to 157.14)	42.58 (-18.78 to 138.34)	13.45 (-313.44 to 831.86)	57.67 (7.88 – 133.49)
Ningxia	46.66 (-62.17 to 452.69)	30.13 (-178.72 to 847.42)	39.59 (-79.73 to 520.10)	37.72 (-71.66 to 464.93)	47.77 (-52.93 to 409.74)	11.36 (-666.99 to 914.94)	48.39 (-34.95 to 226.24)
Guangxi	46.02 (-0.19 to 112.43)	30.59 (-35.04 to 192.07)	34.72 (-413.78 to 915.18)	39.29 (-27.45 to 166.61)	39.09 (-4.41 to 111.20)	16.50 (-1010.02 to 1519.91)	42.42 (-1.95 to 113.08)
Guangdong	32.63 (-10.94 to 98.23)	7.07 (-59.26 to 134.98)	42.58 (-307.77 to 750.74)	34.81 (-38.26 to 191.40)	32.43 (-10.36 to 92.30)	26.04 (-843.98 to 994.86)	31.52 (-9.61 to 98.25)
Yunnan	35.31 (-66.59 to 346.42)	16.64 (-147.27 to 805.61)	25.55 (-76.23 to 468.80)	29.73 (-73.67 to 462.71)	25.55 (-60.15 to 253.56)	9.71 (-722.01 to 667.08)	31.96 (-42.82 to 211.71)
Inner Mongolia	11.71 (-23.32 to 60.43)	3.91 (-449.40 to 425.03)	14.17 (-1351.17 to 1113.27)	10.07 (-40.01 to 96.11)	14.69 (-31.10 to 95.54)	-12.48 (-493.77 to 463.31)	24.76 (-15.07 to 83.76)
Hainan	10.87	-7.20	7.44	7.85	6.31	-4.74	10.58

Parameter	Cause-specific			Sex		Age (years)	
	**Cardiovascular diseases**	**Respiratory diseases**	**Other diseases**	**Male**	**Female**	**0 − 64**	**≥ 65**
	(-25.47 to 66.18)	(-62.21 to 120.38)	(-329.46 to 512.06)	(-43.83 to 117.72)	(-29.42 to 58.25)	(-742.28 to 786.69)	(-24.74 to 65.31)
Liaoning	-3.07<br>(-32.82 to 38.21)	-12.94<br>(-562.15 to 642.01)	-0.05<br>(-889.56 to 614.56)	-2.88<br>(-46.14 to 79.38)	-1.98<br>(-40.37 to 67.17)	-19.19<br>(-179.58 to 473.77)	9.37<br>(-23.63 to 61.57)
Heilongjiang	-1.30<br>(-29.56 to 35.36)	-24.25<br>(-326.25 to 421.62)	-0.89<br>(-1526.99 to 824.05)	-4.26<br>(-45.91 to 75.98)	-0.97<br>(-40.90 to 64.36)	-25.01<br>(-472.25 to 464.55)	11.41<br>(-26.20 to 67.91)
Jilin	-27.31<br>(-48.89 to 2.46)	-42.52<br>(-228.10 to 318.42)	-26.20<br>(-1120.61 to 680.92)	-28.68<br>(-62.70 to 27.17)	-26.62<br>(-56.49 to 22.65)	-43.13<br>(-257.01 to 211.49)	-17.09<br>(-44.13 to 26.66)

CI confidence interval

Table S9 Percentage change [% (95% CI)] in the AF of deaths attributable to heatwaves in 2022 compared to the average during 2000 − 2021, causes of death, sex, and age

Parameter	Cause-specific			Sex		Age (years)	
	**Cardiovascular diseases**	**Respiratory diseases**	**Other diseases**	**Male**	**Female**	**0 − 64**	**≥ 65**
**National**	58.77 (33.38 – 90.76)	76.19 (19.42 – 157.43)	73.79 (8.84 – 190.89)	63.00 (23.77 – 114.77)	65.57 (65.24 – 65.94)	63.99 (-16.17 to 251.76)	64.56 (36.93 – 97.13)
**Provincial level**							
Xizang	157.85 (-30.52 to 707.85)	164.21 (-748.74 to 1699.40)	161.27 (-73.07 to 1177.76)	159.04 (-43.12 to 979.21)	160.65 (-11.82 to 778.23)	161.75 (-2638.65 to 1948.28)	158.24 (20.02 – 484.69)
Sichuan	133.26 (-34.07 to 746.07)	133.68 (-310.33 to 1638.43)	133.96 (-73.83 to 1196.94)	133.45 (-57.89 to 696.44)	133.93 (-32.04 to 682.04)	137.24 (-1414.40 to 1735.69)	132.90 (13.38 – 437.20)
Chongqing	120.60 (-41.45 to 722.89)	119.65 (-881.92 to 1675.98)	120.12 (-71.16 to 771.13)	120.06 (-53.87 to 700.07)	120.44 (-34.39 to 572.53)	121.42 (-1147.08 to 2036.50)	120.74 (-8.01 to 395.52)
Guizhou	115.70 (41.83 – 221.62)	117.39 (13.17 – 343.48)	115.55 (-671.13 to 1322.87)	115.38 (12.46 – 327.57)	116.66 (44.59 – 209.58)	121.77 (-1991.60 to 1800.76)	113.32 (47.02 – 214.46)
Zhejiang	116.03 (61.14 – 182.56)	114.74 (17.83 – 253.49)	115.28 (45.60 – 218.79)	115.45 (64.05 – 187.76)	115.55 (71.97 – 165.89)	116.72 (36.82 – 235.61)	115.23 (82.27 – 155.77)
Hunan	114.62 (46.39 – 218.34)	114.02 (0.81 – 312.45)	113.81 (-719.99 to 1838.19)	114.04 (4.86 – 347.18)	114.50 (46.03 – 211.04)	116.37 (-1103.14 to 2433.95)	112.54 (44.06 – 211.51)
Qinghai	108.81 (-40.04 to 630.74)	112.09 (-422.69 to 1183.80)	111.20 (-72.25 to 1079.33)	109.96 (-71.81 to 764.00)	110.80 (-32.79 to 551.02)	112.40 (-1108.85 to 2115.25)	109.77 (-13.13 to 374.69)
Jiangxi	99.46 (36.87 – 199.63)	99.70 (-4.68 to 334.06)	99.33 (-580.55 to 1695.95)	99.35 (-2.73 to 306.87)	99.61 (33.49 – 200.07)	100.84 (-1685.58 to 2468.41)	98.30 (35.20 – 197.29)
Jiangsu	98.44 (50.14 – 156.62)	98.17 (15.90 – 221.23)	98.25 (31.11 – 194.90)	98.14 (50.54 – 164.28)	98.50 (59.58 – 145.10)	99.94 (26.82 – 214.03)	99.38 (69.31 – 135.81)
Henan	95.68	103.84	98.67	97.82	97.12	102.78	99.24

Parameter	Cause-specific			Sex		Age (years)	
	Cardiovascular diseases	Respiratory diseases	Other diseases	Male	Female	0 − 64	≥ 65
	(37.04 – 181.59)	(-264.54 to 1151.46)	(-2740.21 to 2158.79)	(5.54 – 260.94)	(15.87 – 242.51)	(-380.25 to 1456.38)	(36.18 – 199.06)
Hubei	97.17<br>(32.06 – 203.04)	96.90<br>(-9.02 to 299.88)	96.87<br>(-815.69 to 1440.81)	96.99<br>(-3.18 to 270.64)	97.06<br>(26.93 – 191.26)	99.29<br>(-2057.36 to 1803.07)	98.23<br>(33.75 – 195.09)
Anhui	91.55<br>(49.20 – 149.59)	93.49<br>(6.56 – 213.03)	92.66<br>(28.73 – 183.16)	92.19<br>(43.32 – 159.90)	92.31<br>(52.03 – 135.21)	94.33<br>(27.99 – 195.08)	93.31<br>(64.35 – 128.89)
Fujian	91.00<br>(27.84 – 190.54)	92.20<br>(-25.61 to 338.29)	90.16<br>(-595.70 to 1459.13)	90.80<br>(-6.19 to 268.55)	90.62<br>(26.87 – 195.85)	93.33<br>(-1335.89 to 1355.57)	90.38<br>(33.64 – 191.46)
Shanghai	89.92<br>(46.04 – 148.84)	90.35<br>(-0.81 to 227.05)	90.69<br>(29.33 – 174.65)	90.34<br>(40.36 – 152.04)	90.38<br>(51.88 – 136.00)	89.90<br>(23.04 – 197.19)	91.29<br>(62.54 – 124.96)
Gansu	81.29<br>(-53.87 to 539.60)	80.94<br>(-441.28 to 1404.21)	80.74<br>(-65.16 to 562.91)	81.01<br>(-67.49 to 538.71)	81.07<br>(-38.96 to 528.27)	78.48<br>(-232.66 to 1017.84)	80.45<br>(-22.02 to 303.56)
Xinjiang	56.12<br>(-47.60 to 440.43)	61.21<br>(-578.13 to 1074.15)	57.04<br>(-85.89 to 701.62)	57.49<br>(-74.41 to 442.85)	56.84<br>(-51.44 to 389.98)	58.81<br>(-689.08 to 1336.69)	56.27<br>(-32.84 to 251.90)
Shaanxi	56.18<br>(-53.38 to 585.36)	51.48<br>(-690.66 to 835.49)	54.93<br>(-88.31 to 663.58)	55.26<br>(-72.65 to 549.43)	55.15<br>(-49.44 to 355.68)	52.47<br>(-882.49 to 928.87)	55.98<br>(-30.25 to 233.80)
Ningxia	47.51<br>(-61.95 to 455.87)	46.62<br>(-188.70 to 967.42)	46.97<br>(-78.66 to 552.88)	47.05<br>(-69.74 to 503.22)	47.42<br>(-53.04 to 408.57)	45.71<br>(-841.88 to 1228.00)	45.60<br>(-36.17 to 220.11)
Guangdong	46.26<br>(-1.79 to 118.61)	50.30<br>(-42.81 to 229.84)	45.65<br>(-312.25 to 769.06)	46.36<br>(-32.97 to 216.36)	46.71<br>(-0.69 to 113.04)	47.44<br>(-970.32 to 1180.79)	46.01<br>(0.35 – 120.10)
Shandong	36.75<br>(2.41 – 84.32)	41.40<br>(-27.10 to 155.89)	38.37<br>(-10.10 to 102.90)	37.26<br>(3.25 – 85.24)	38.64<br>(12.01 – 68.52)	40.44<br>(-12.26 to 121.32)	38.71<br>(17.37 – 62.99)
Beijing	37.43<br>(-5.90 to 101.59)	38.76<br>(-606.36 to 869.76)	37.40<br>(-1247.94 to 1956.25)	37.20<br>(-23.40 to 146.24)	37.89<br>(-18.74 to 126.06)	38.06<br>(-876.41 to 950.25)	39.93<br>(-2.57 to 111.86)

Parameter	Cause-specific			Sex		Age (years)	
	Cardiovascular diseases	Respiratory diseases	Other diseases	Male	Female	0 − 64	≥ 65
Guangxi	34.34<br>(-8.17 to 95.44)	36.26<br>(-32.22 to 204.76)	35.80<br>(-416.30 to 923.31)	35.18<br>(-29.59 to 158.74)	35.29<br>(-7.01 to 105.43)	37.34<br>(-1172.79 to 1809.64)	33.70<br>(-7.95 to 100.04)
Shanxi	30.77<br>(-8.32 to 85.12)	31.15<br>(-422.93 to 751.67)	30.72<br>(-1438.95 to 1647.93)	30.83<br>(-28.88 to 136.62)	30.72<br>(-25.53 to 118.51)	30.94<br>(-346.37 to 975.60)	30.70<br>(-10.57 to 93.56)
Hebei	27.45<br>(-10.16 to 84.14)	31.35<br>(-277.83 to 789.75)	28.92<br>(-950.28 to 1080.93)	28.03<br>(-30.44 to 145.46)	28.56<br>(-27.73 to 115.00)	29.60<br>(-499.98 to 890.82)	29.41<br>(-11.06 to 99.17)
Tianjin	27.02<br>(-12.57 to 84.78)	29.23<br>(-649.68 to 1028.72)	26.37<br>(-1551.54 to 1432.07)	26.05<br>(-30.58 to 132.70)	27.93<br>(-24.46 to 125.07)	27.81<br>(-401.86 to 869.19)	28.67<br>(-12.48 to 95.13)
Yunnan	16.13<br>(-71.33 to 283.14)	20.77<br>(-148.94 to 837.64)	20.43<br>(-77.20 to 445.59)	18.02<br>(-76.04 to 411.93)	19.60<br>(-62.04 to 236.80)	24.56<br>(-806.16 to 770.86)	17.14<br>(-49.24 to 176.70)
Hainan	1.01<br>(-32.10 to 51.41)	10.12<br>(-55.16 to 161.50)	3.72<br>(-321.52 to 490.87)	3.35<br>(-46.17 to 108.65)	3.30<br>(-31.42 to 53.78)	7.54<br>(-825.09 to 901.02)	1.57<br>(-30.87 to 51.83)
Inner Mongolia	1.82<br>(-30.11 to 46.23)	2.64<br>(-445.13 to 418.61)	2.01<br>(-1217.93 to 984.07)	2.02<br>(-44.40 to 81.76)	1.90<br>(-38.78 to 73.74)	1.46<br>(-556.51 to 553.06)	1.75<br>(-30.74 to 49.87)
Liaoning	-13.61<br>(-40.12 to 23.18)	-8.41<br>(-586.18 to 680.60)	-12.88<br>(-788.22 to 522.84)	-13.08<br>(-51.79 to 60.55)	-12.88<br>(-47.00 to 48.59)	-11.68<br>(-186.98 to 527.10)	-13.39<br>(-39.53 to 27.93)
Heilongjiang	-17.23<br>(-40.93 to 13.52)	-16.06<br>(-350.70 to 477.99)	-17.03<br>(-1294.65 to 673.60)	-17.06<br>(-53.14 to 52.46)	-17.06<br>(-50.50 to 37.66)	-17.27<br>(-510.63 to 522.76)	-18.01<br>(-45.69 to 23.58)
Jilin	-37.87<br>(-56.31 to -12.43)	-35.32<br>(-244.14 to 370.81)	-37.61<br>(-962.72 to 560.11)	-37.60<br>(-67.36 to 11.28)	-37.66<br>(-63.04 to 4.19)	-36.71<br>(-274.73 to 246.65)	-38.26<br>(-58.39 to -5.68)

AF attributable fractions, *CI* confidence interval

Table S10 The sensitivity analyses of the ER, AN, and AF

Sensitivity analysis specifications	ER [% (95% CI)]	AN in 2022 (95% CI)	AF in 2022 [‰ (95% CI)]
Changing the definition of heatwave			
HW900.2	7.00 (6.18 – 7.83)	63,281 (55,220 – 71,026)	18.20 (15.88 – 20.43)
HW900.3	6.98 (6.14 – 7.83)	54,221 (46,651 – 61,767)	15.59 (13.36 – 17.76)
HW900.4	7.04 (6.21 – 7.87)	47,833 (40,873 – 55,021)	13.76 (11.75 – 15.82)
HW900.5	6.90 (6.05 – 7.76)	42,545 (35,828 – 49,247)	12.24 (10.30 – 14.16)
HW925.2	8.41 (7.44 – 9.39)	62,961 (54,945 – 70,413)	18.11 (15.80 – 20.25)
HW925.3	8.09 (7.13 – 9.07)	51,420 (44,522 – 58,367)	14.79 (12.80 – 16.79)
HW925.4	7.83 (6.82 – 8.86)	41,912 (35,233 – 48,029)	12.05 (10.13 – 13.81)
HW925.5	8.01 (6.94 – 9.09)	39,063 (32,461 – 45,317)	11.23 (9.34 – 13.03)
HW950.2	9.6 0(8.44 – 10.77)	55,069 (47,906 – 62,269)	15.84 (13.78 – 17.91)
HW950.3	9.27 (8.01 – 10.55)	45,691 (38,757 – 52,654)	13.14 (11.15 – 15.14)
HW950.4	9.50 (8.18 – 10.83)	39,591 (33,303 – 45,938)	11.39 (9.58 – 13.21)
Changing the maximum lag day			
1	7.64 (6.83 – 8.45)	57,025 (50,630 – 63,482)	16.40 (14.56 – 18.26)
2	8.37 (7.48 – 9.26)	63,131 (55,578 – 69,829)	18.16 (15.98 – 20.08)
3	8.41 (7.44 – 9.39)	62,961 (54,945 – 70,413)	18.11 (15.80 – 20.25)
4	8.41 (7.36 – 9.47)	62,881 (54,496 – 71,449)	18.08 (15.67 – 20.55)
5	8.03 (6.91 – 9.16)	59,235 (50,309 – 67,718)	17.04 (14.47 – 19.47)
Changing the *df* of time variable			
3/year	7.97 (7.02 – 8.94)	59,043 (55,197 – 69,268)	16.98 (15.87 – 19.92)
4/year	8.41 (7.44 – 9.39)	62,961 (54,945 – 70,413)	18.11 (15.80 – 20.25)
5/year	8.54 (7.57 – 9.53)	65,600 (62,240 – 77,762)	18.87 (17.90 – 22.36)
Adjusting for environmental covariates			
+ RH + $PM_{2.5}$ + O_3	8.41 (7.44 – 9.39)	62,961 (54,945 – 70,413)	18.11 (15.80 – 20.25)
+ RH + $PM_{2.5}$	8.65 (7.70 – 9.60)	65,707 (58,245 – 72,895)	18.90 (16.75 – 20.96)
+ RH + O_3	8.38 (7.40 – 9.36)	63,534 (55,602 – 71,154)	18.27 (15.99 – 20.46)
+ RH	8.70 (7.77 – 9.65)	66,944 (59,504 – 74,065)	19.25 (17.11 – 21.30)
+ $PM_{2.5}$ + O_3	8.36 (7.42 – 9.31)	63,861 (56,724 – 71,056)	18.37 (16.31 – 20.43)
+ $PM_{2.5}$	9.21 (8.24 – 10.18)	70,682 (63,212 – 78,103)	20.33 (18.18 – 22.46)
+ O_3	8.36 (7.42 – 9.31)	64,416 (56,801 – 71,927)	18.53 (16.34 – 20.69)
Altering the heatwave temperature indicator			
Daily maximum temperature	8.41 (7.44 – 9.39)	62,961 (54,945 – 70,413)	18.11 (15.80 – 20.25)
Daily minimum temperature	5.87 (5.02 – 6.73)	42,403 (35,174 – 49,558)	12.19 (10.12 – 14.25)
Heat index	7.03 (6.22 – 7.84)	57,642 (50,499 – 65,294)	16.58 (14.52 – 18.78)
Mortality rate in 2022			
Estimated mortality rate based on trends	8.41 (7.44 – 9.39)	60,640 (52,662 – 68,853)	18.13 (15.74 – 20.58)
Constant mortality rate	8.41 (7.44 – 9.39)	62,961 (54,945 – 70,413)	18.11 (15.80 – 20.25)
Year division			
2006 – 2017	8.41 (7.44 – 9.39)	/	/
2006 – 2011	8.92 (7.15 – 10.48)	/	/
2013 – 2017	8.28 (7.22 – 9.67)	/	/

ER excess risk, *AN* attributable number, *AF* attributable fraction, *df* degrees of freedom, *RH* relative humidity

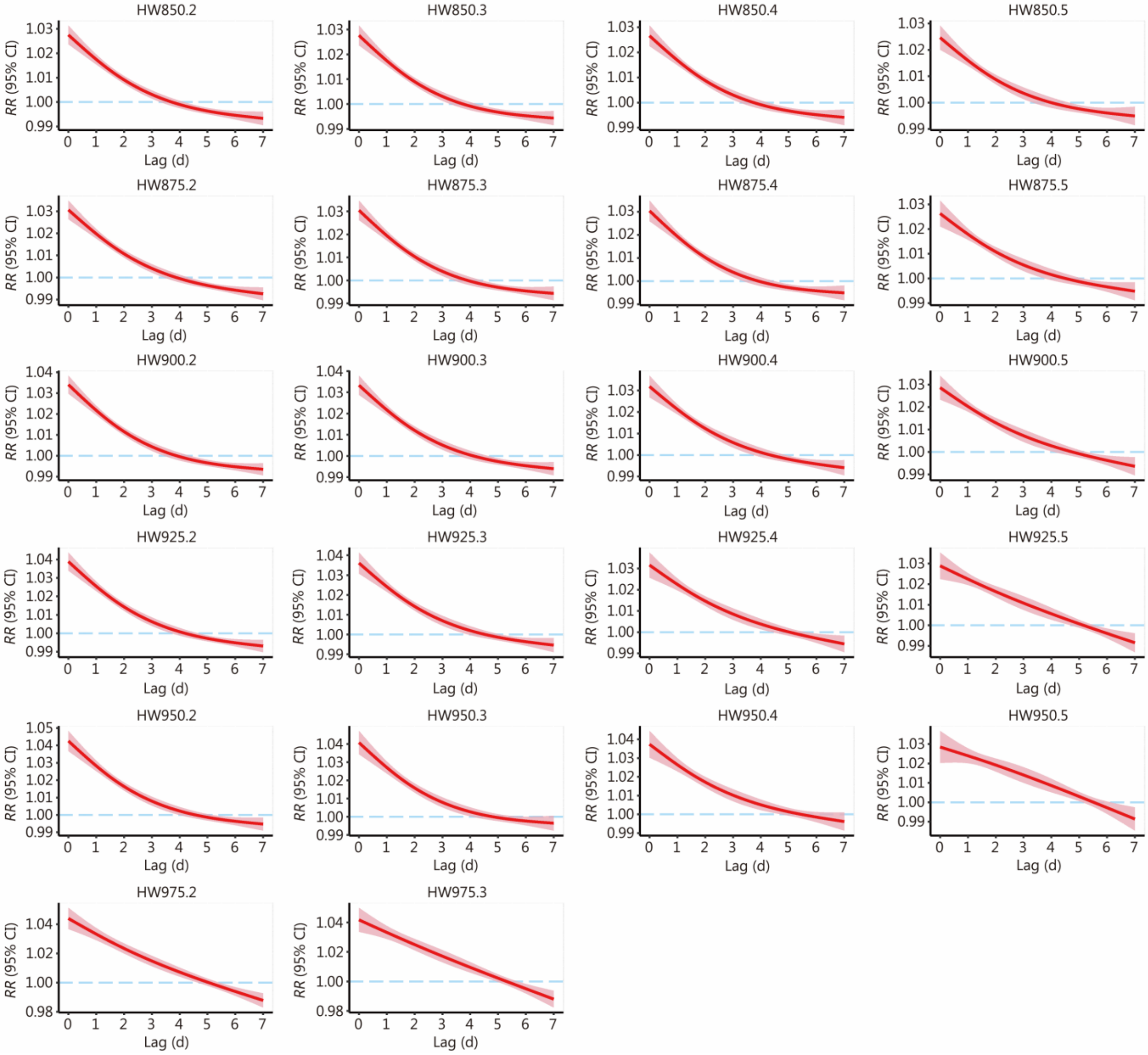

Fig. S1 The lag patterns in the effects of various heatwave definitions on non-accidental mortality. The lag effects of heatwaves on non-accidental mortality were estimated by a distributed lag nonlinear model (DLNM). The red solid line represents the effect (*RR*, 95% CI) of heatwaves on non-accidental mortality. The horizontal blue dashed line is the reference line (*RR* = 1). HW heatwaves, *RR* relative risk, CI confidence interval

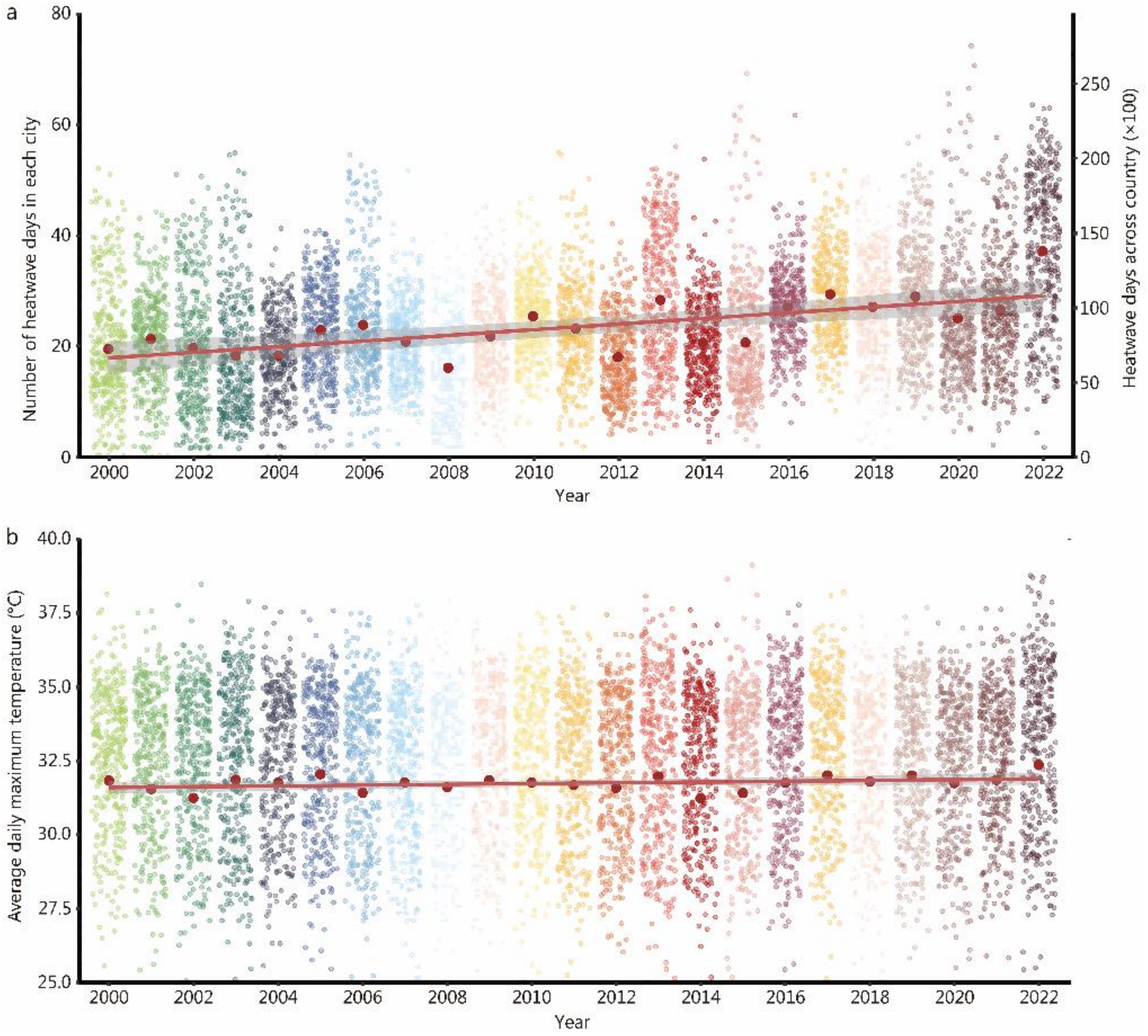

Fig. S2 The characteristics of heatwaves and average daily maximum temperature in 2022 compared with those during 2000 – 2021 in China. **a** Temporal trend of heatwaves from 2000 to 2022. **b** Temporal trend of average daily maximum temperature during heatwave in each city and in China from 2000 to 2022. The temporal changes of number of heatwaves and the average daily maximum temperature were fitted by a generalized linear model

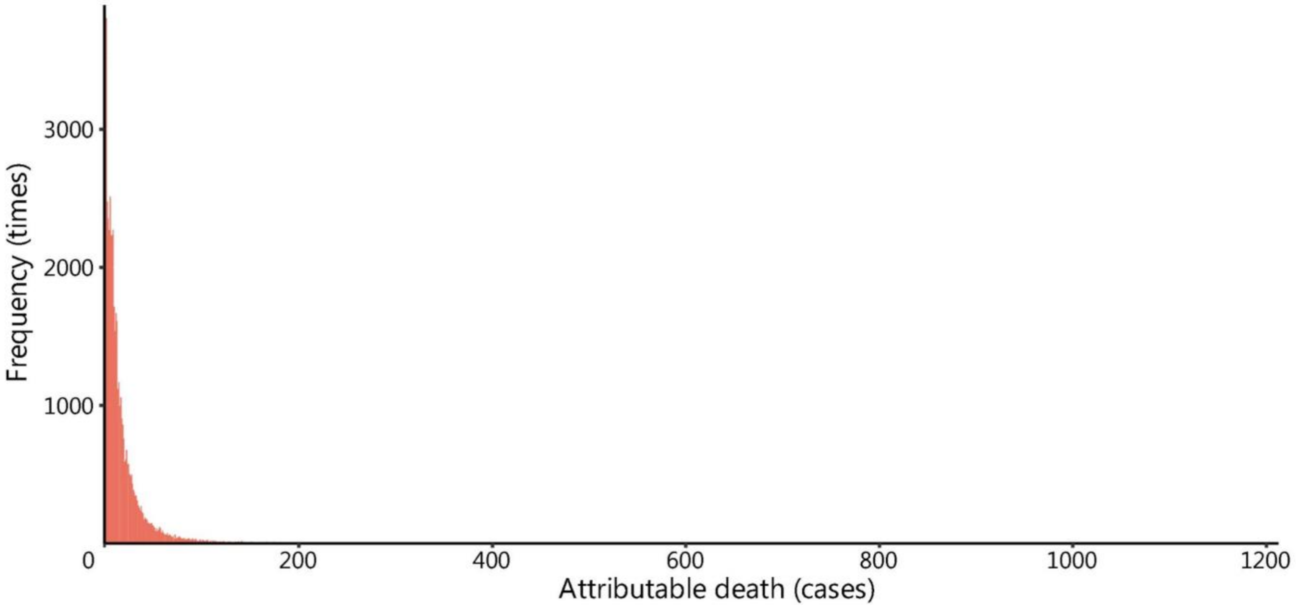

Fig. S3 The frequency distribution of attributable deaths per heatwave event in each city of China during 2000 – 2022

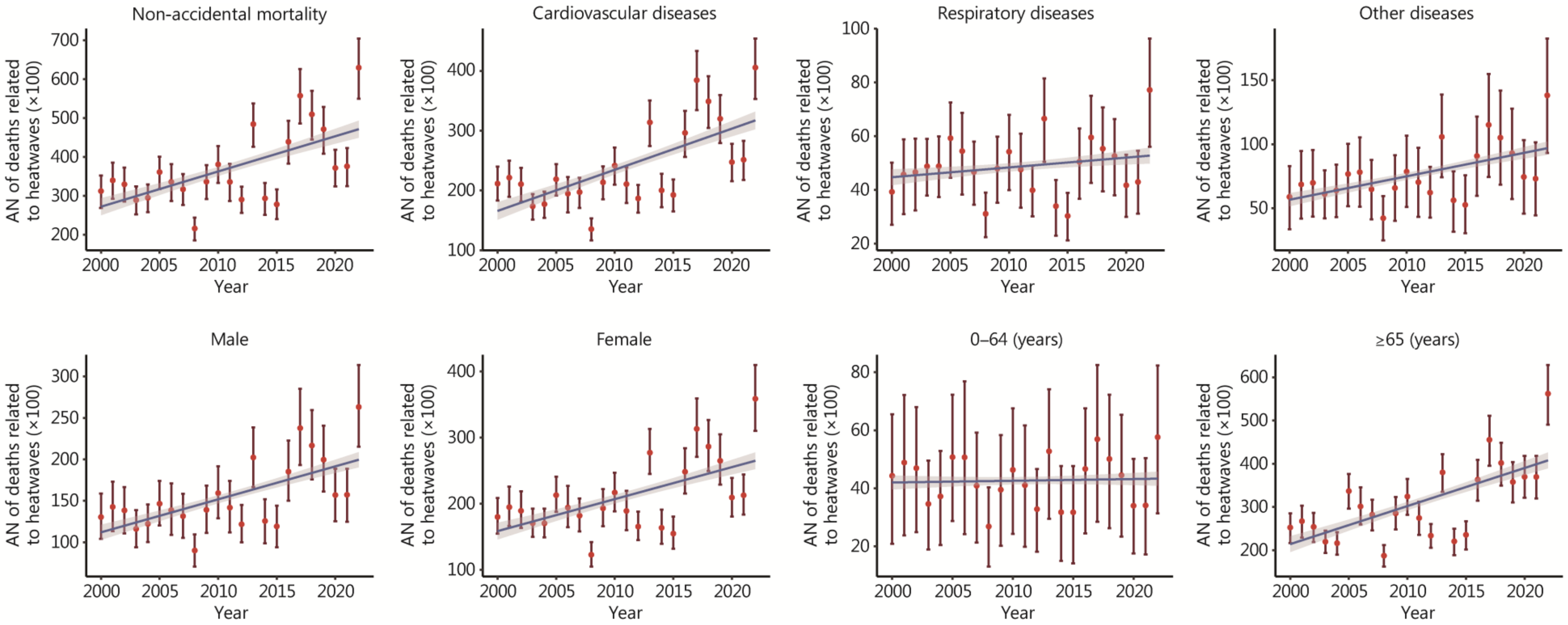

Fig. S4 The trend of AN of deaths related to heatwaves in China, 2000 – 2022. The red dots indicate the number of deaths attributable to heatwaves from 2000 – 2022, and the red error line indicates the 95% CI for heatwave-attributable deaths. The solid blue line indicates the trend of the linear fit (general linear regression) to the heatwave-attributable deaths from 2000 – 2022, and the gray shading indicates the 95% CI. CI confidence internal

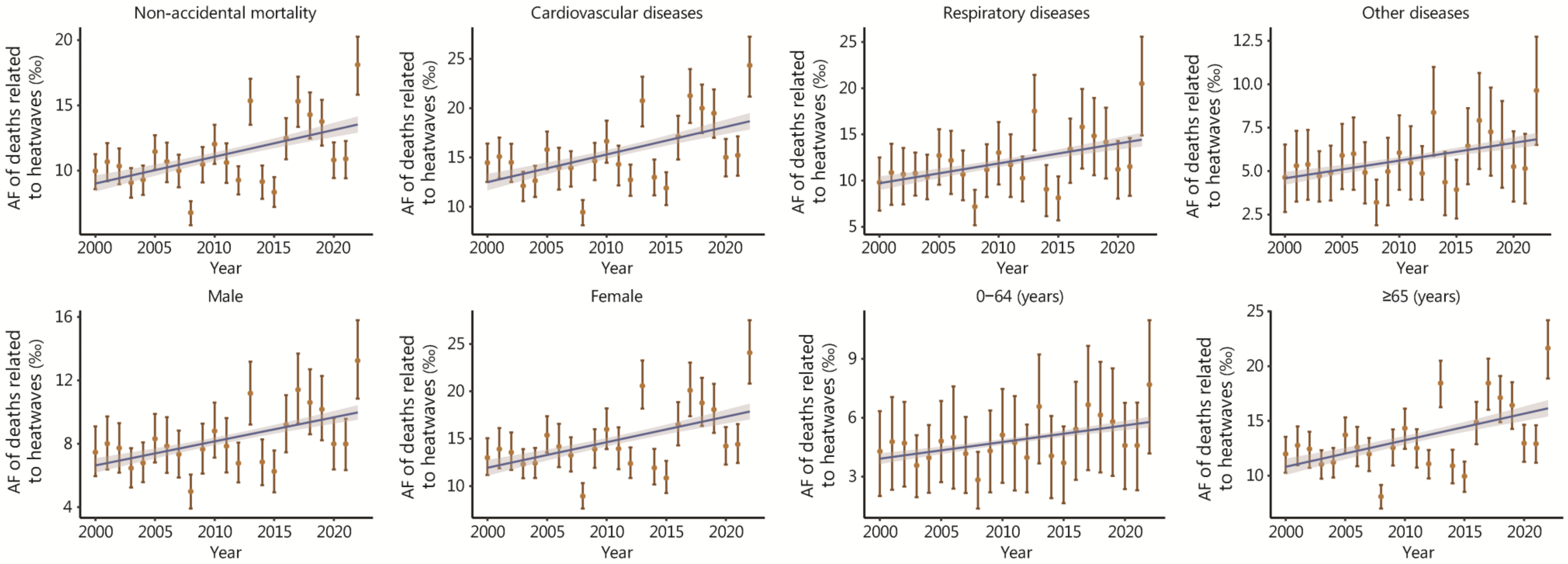

Fig. S5 The trend of AF of death related to heatwaves in China, 2000 – 2022. The red dots indicate the attributable fractions from 2000 – 2022, and the red error line indicates the 95% CI for heatwave-attributable fractions. The solid blue line indicates the trend of the linear fit (general linear regression) to the heatwave-attributable fractions from 2000 – 2022, and the gray shading indicates the 95% CI. AF attributable fraction, CI confidence interval

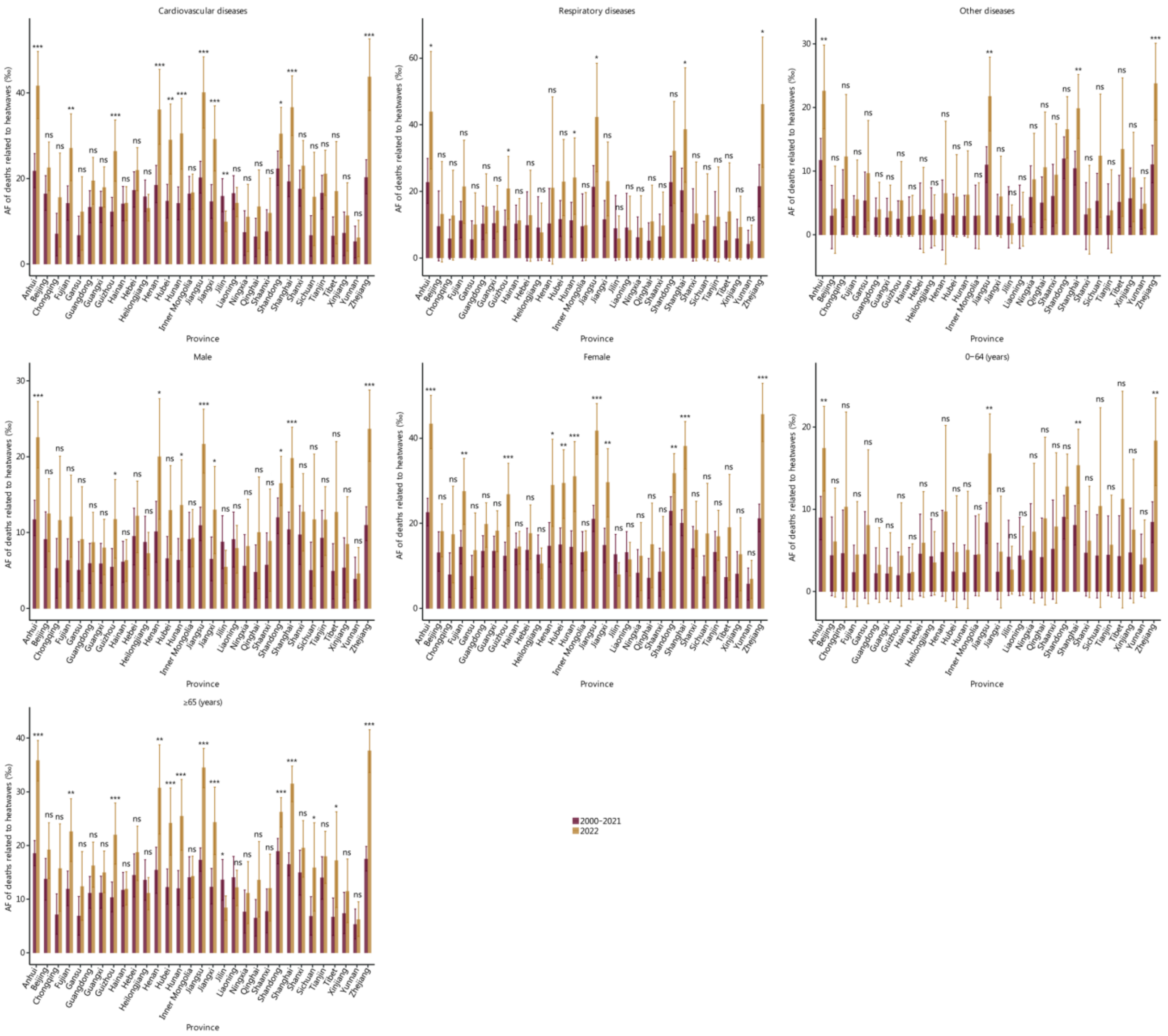

Fig. S6 Comparison of the AF of death related to heatwaves in 2022 with that during 2000 – 2021 by sex, age, and cause of death. ns non-significant, $^{*}P < 0.05$, $^{**}P < 0.01$, $^{***}P < 0.001$, AF attributable fraction

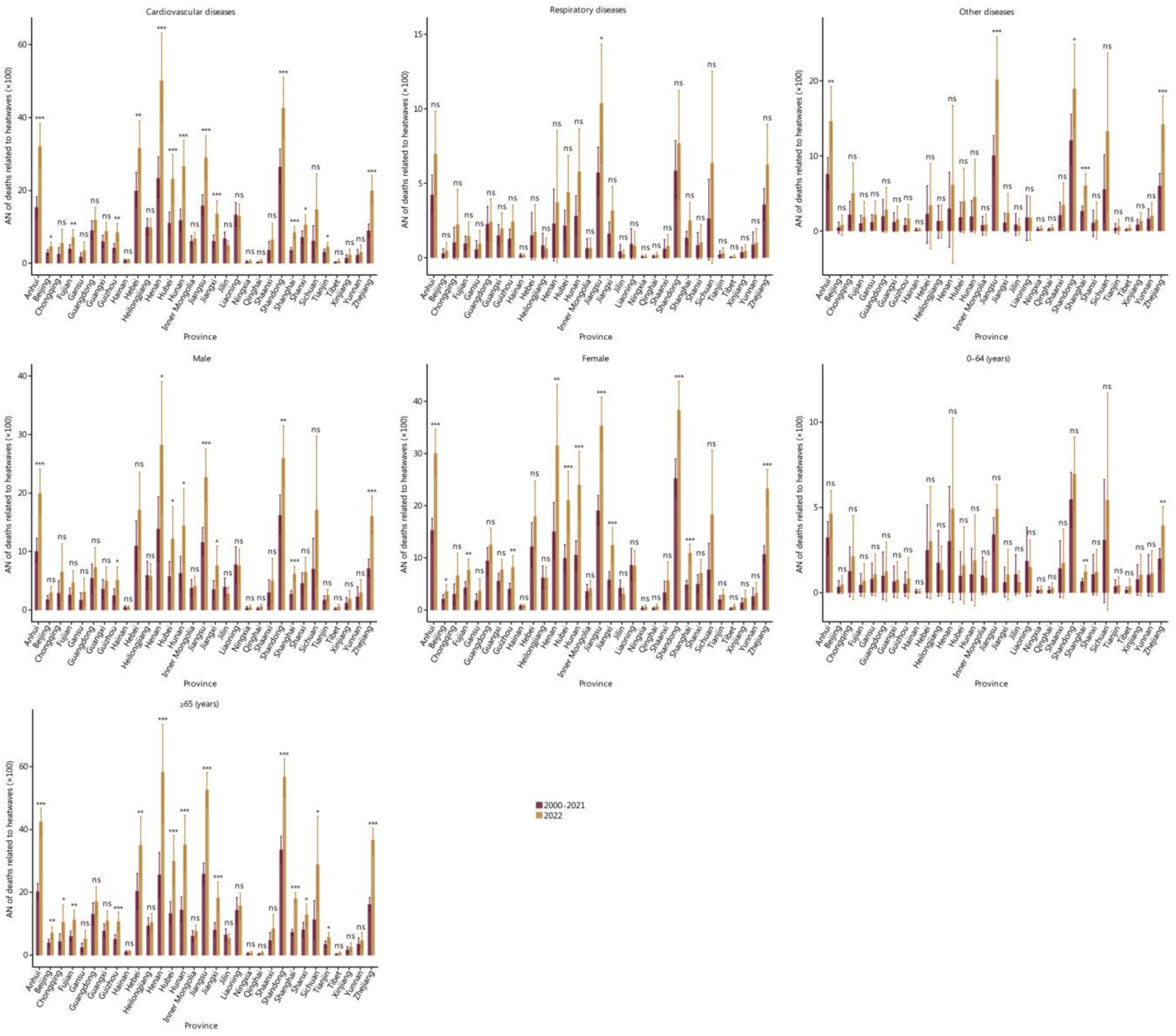

Fig. S7 Comparison of the AN of death related to heatwaves in 2022 with that during 2000 – 2021 by sex, age, and cause of death. ns non-significant, $^{*}P < 0.05$, $^{**}P < 0.01$, $^{***}P < 0.001$
